# Mitigation Effect of Exogenous Nano-Silicon on Salt Stress Damage of Rice Seedlings

**DOI:** 10.3390/ijms26010085

**Published:** 2024-12-25

**Authors:** Jian Xiong, Xiaohui Yang, Minmin Sun, Jianqin Zhang, Linchong Ding, Zhiyuan Sun, Naijie Feng, Dianfeng Zheng, Liming Zhao, Xuefeng Shen

**Affiliations:** 1 College of Coastal Agricultural Sciences, Guangdong Ocean University, Zhanjiang 524008, China; 2112204010@stu.gdou.edu.cn (J.X.); 2112204049@stu.gdou.edu.cn (X.Y.); 2112204037@stu.gdou.edu.cn (M.S.); 21122040151@stu.gdou.edu.cn (J.Z.); dinglinchong0405@163.com (L.D.); sunzhiyuan@hndx2.wecom.work (Z.S.); fengnj@gdou.edu.cn (N.F.); zhengdf@gdou.edu.cn (D.Z.); nxyzlm@gdou.edu.cn (L.Z.); 2 National Saline-Tolerant Rice Technology Innovation South China Center, Zhanjiang 524008, China

**Keywords:** rice, salt stress, nano-silicon, antioxidant system, endogenous hormones, ion balance

## Abstract

Salt stress represents a significant abiotic stress factor that impedes the growth of rice. Nano-silicon has the potential to enhance rice growth and salt tolerance. In this experiment, the rice variety 9311 was employed as the test material to simulate salt stress via hydroponics, with the objective of investigating the mitigation effect of foliar application of nano-silicon on rice seedlings. The results demonstrated that NaCl stress markedly impeded the growth of rice seedlings after seven days of NaCl treatment. The foliar application of nano-silicon followed by NaCl stress alleviated the growth of rice seedlings, markedly improved the spatial conformation of the root system, and enhanced photosynthesis compared with that of NaCl stress alone. The activities of antioxidant enzymes were improved. The contents of antioxidants were increased, and the over-accumulation of ROS was reduced. Furthermore, the foliar application of nano-silicon was found to enhance the uptake of Si^4+^, K^+^, and Ca^2+^ in plants, while simultaneously reducing Na^+^ and Cl^−^ accumulation. Additionally, the content of IAA, CTK, GA, JA, and SA was increased, and ABA was decreased. In conclusion, the foliar application of nano-silicon has been demonstrated to alleviate salt stress injury and improve the growth of rice seedlings.

## 1. Introduction

Soil salinization is one of the major abiotic stresses limiting food production. Saline soil areas in China are reaching 1.00 × 10^8^ hectares, over half of which is suited for rice (*Oryza sativa* L.) cultivation [1]. Rice, a moderately salt-sensitive crop, plays a significant role in China’s food crop production [2]. The seedling period is the stage of reproduction during which the plant is most susceptible to salt damage, with the greatest impact on morphogenesis and, consequently, on rice yield and quality [3]. Additionally, pot and field experiments have demonstrated that salt stress impairs seedling density and seedling biomass in rice [4]. The development and implementation of salt-tolerant rice cultivation strategies can improve the growth and development of rice seedlings, enhance rice yield, and increase quality; further, nanobiotechnology has great developmental potential in this field [5].

It has been found that salt stress can lead to crop yield reductions of up to 40% due to various environmental factors [6], with sodium chloride (NaCl) being one of the primary components affecting salt stress [7]. Salt stress disrupts osmotic balance and ionic homeostasis in plants, inducing secondary stress that leads to the accumulation of reactive oxygen species (ROS), such as hydrogen peroxide (H_2_O_2_) and superoxide anion (O_2_^·−^). This accumulation results in oxidative damage to plant cells, affecting the growth and development of rice seedlings, which manifests as stunted plant growth, yellowing and wilting of leaves, and inhibited root development [8]. Malondialdehyde (MDA), a product of lipid peroxidation in cell membranes, can be measured to assess the degree of oxidative damage to plants [9]. Plants have evolved an antioxidant defense system to scavenge the excessive accumulation of ROS. This system is divided into two types: antioxidant enzymes and non-enzymatic antioxidants. The enzymatic antioxidant system comprises superoxide dismutase (SOD), peroxidase (POD), catalase (CAT), and ascorbate–glutathione cycle-related enzymes, including ascorbate reductase (APX), glutathione reductase (GR), dehydroascorbate reductase (DHAR), and monodehydroascorbate reductase (MDHAR) [10]. Non-enzymatic antioxidants include reduced glutathione (GSH), oxidized glutathione (GSSG), ascorbic acid (AsA), and dehydroascorbic acid (DHA) [11]. As the duration of the stress period increases, the transpiration rate declines, the net photosynthetic rate is suppressed, and photosynthetic pigment degradation may even occur [12]. An excessive concentration of salt ions in the growth environment will rapidly reduce the osmotic potential of the plant growth environment, thereby causing a physiological water deficit in the plant [13]. NaCl is one of the major constituents of salt stress, and excess Na^+^ in the environment competes for K^+^ uptake sites in the root system, accumulating excess Na^+^ in the plant. This results in a Na^+/^K^+^ imbalance, thus affecting the plant’s growth and metabolism [14].

Silicon (Si) is regarded as one of the most significant beneficial elements for plant life activities, exhibiting no detrimental effects at elevated concentrations [15], and studies have shown that it can enhance the salt tolerance of various crops, including soybean [16], corn [17], and rice [18]. Silicon supplementation helps plants retain water, enhances cell strength, can make stems more erect, and increases chlorophyll content and the rate of photosynthesis. The amount of effective silicon in the environment that can be absorbed and utilized by plants is generally very low, so silicon research is mostly limited to silica-loving crops [19]. Silicon is a key element for rice plants, which are often used as a benchmark in silicon nutrition studies. New research suggests that using silicon fertilizers can mitigate the negative impacts of salt stress on the development of rice seedlings [20]. Silica nanoparticles (NPs-Si) represent one of the applications of nanobiotechnology, and researchers have conducted numerous studies due to their unique physicochemical properties, eco-friendliness, and non-toxicity. Because of its small size and strong penetrating ability, nano-silicon can more effectively alleviate environmental stresses on plants compared to bulk silicon, such as by improving plant growth under salt stress [21]. Badawy demonstrated that the exogenous application of nano-silicon to rice plants subjected to salt stress resulted in enhanced water content and ion selectivity within the plants [22]. Riaz showed that the exogenous application of nano-silicon could enhance the resistance of rice seedlings by increasing the activity of antioxidant enzymes (SOD, CAT, and POD) and increasing the content of antioxidants (AsA and GSH) [23]. Ijaz observed that the application of exogenous nano-silicon resulted in an increase in the content of photosynthetic pigments and a reduction in the adverse effects of salt stress on rice seedlings [24]. The exogenous application of nano-silicon has been demonstrated to increase the content of osmoregulatory substances in rice, which has resulted in an improvement in growth status and antioxidant enzyme system activity [25]. The exogenous application of nano-silicon has been demonstrated to maintain beneficial element uptake and improve plant growth and development under salt stress conditions by regulating Na^+^/Cl^−^ and Na^+^/K^+^ ratios [26]. Furthermore, Li et al. showed that nano-silicon has the ability to protect the ultrastructural integrity of the cell, thereby maintaining the normal functioning of the organelles [27]. These findings suggest that nano-silicon has the potential to improve the growth of rice seedlings under salt stress conditions.

The mechanism of action of exogenously applied nano-silicon to improve rice salt tolerance and promote the growth of rice seedlings under salt stress is currently poorly understood, particularly regarding the response mechanism of rice seedlings subjected to salt stress within a short period of time after nano-silicon treatment. The objective of this study is to gain further insight into the mechanisms by which the foliar application of nano-silicon mitigates the adverse effects of salt stress on rice seedlings and enhances morphological growth, photosynthesis, antioxidant regulation, and the reduction in oxidative stress in rice seedlings, providing new insights to explore the regulatory effects of exogenous nano-silicon on salt tolerance in rice.

## 2. Results

### 2.1. Effects of Nano-Silicon on the Above-Ground Morphological Parameters of Rice Seedlings Under NaCl Stress

NaCl stress significantly inhibited the above-ground growth of rice seedlings, and foliar application of nano-silicon could significantly alleviate the inhibitory effect of NaCl. From Figure S1 and Table 1, it can be seen that in comparison to the control (CK), the S treatment notably reduced the plant height by 11.46%, stem diameter at the base by 21.28%, leaf area by 34.04%, shoot fresh weight by 31.50% and shoot dry weight by 23.16% of the above-ground parts of rice seedlings. Compared to the S treatment, the SN treatment resulted in a notable increase in several morphological parameters of the seedlings, including plant height by 4.73%, stem basal width by 13.13%, leaf area by 19.63%, shoot fresh weight by 14.20% and shoot dry weight by 15.07%. The results showed that nano-silicon promoted the above-ground growth of rice seedlings undergoing NaCl stress.

### 2.2. Effects of Nano-Silicon on the Root Parameters of Rice Seedlings Under NaCl Stress

**Figure 1:**
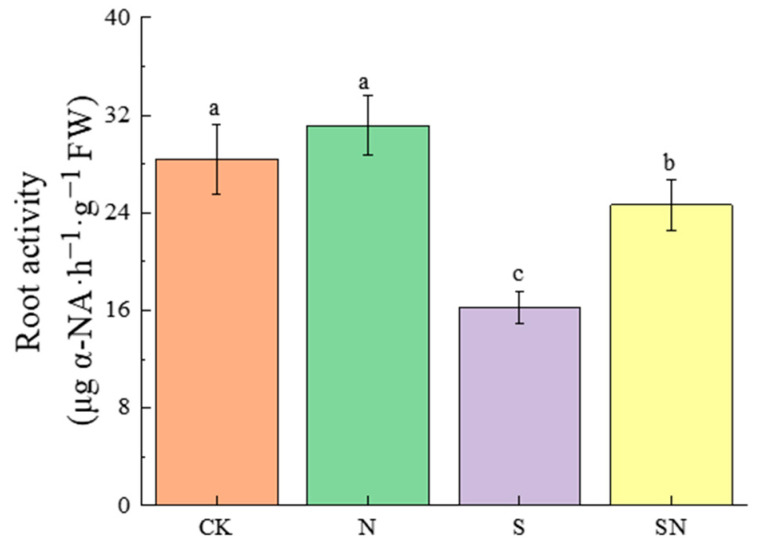
Effects of nano-silicon on root activity of rice seedlings under NaCl stress. (Control (CK), nano-silica treatment (N), salt treatment (S), and nano-silica + salt treatment (SN)). Different lowercase letters indicate significant difference at the 0.05 level among different treatments based on Duncan’s multiple range test.

NaCl stress limits the root growth of rice seedlings, and foliar application of nano-silicon promotes root growth under NaCl stress. Figure S1 and Table 2 illustrate that, relative to CK, the S treatment resulted in a marked decrease in the root length by 11.89%, fresh weight by 30.35%, and dry weight by 35.68% of the seedlings. Compared to the S treatment, the SN treatment significantly increased these root parameters: root length by 10.12%, fresh weight by 19.42%, and dry weight by 25.78%. After 7 d of NaCl stress, compared to CK, the S treatment significantly decreased the total root length by 47.91%, root surface area by 55.47%, root volume by 68.09%, root diameter by 25.99%, root bifurcation number by 57.28%, and root tip number by 51.91%. Relative to the S treatment, the SN treatment markedly increased these root structural parameters: total root length by 68.63%, root surface area by 34.17%, root volume by 38.75%, root diameter by 30.12%, root bifurcation number by 45.81%, and root tip number by 45.91%. Compared to CK, the S treatment led to a considerable reduction in root activity, of 42.86%. Conversely, the SN treatment caused a significant increase in root activity in comparison to the S treatment, of 51.65%, as shown in Table 3 and Figure S2. The results showed that nano-silicon could help rice seedlings by promoting root spatial configuration building and coordinate root growth under NaCl stress (Figure 1).

### 2.3. Effects of Nano-Silica on RWC of Leaves, R/S, and Seedling Index of Rice Seedlings Under NaCl Stress

**Figure 2:**
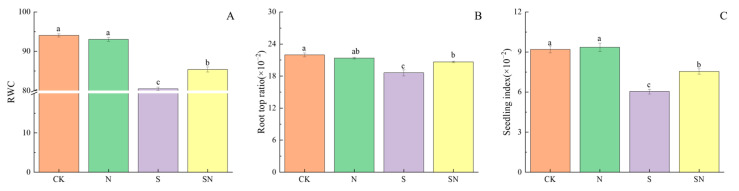
Effects of nano-silicon on RWC, R/S and seedling index of rice seedlings under NaCl stress. (Control (CK), nano-silica treatment (N), salt treatment (S), and nano-silica + salt treatment (SN), RWC (**A**), Root top ratio (**B**), Seedling index (**C**)). Different lowercase letters indicate significant difference at the 0.05 level among different treatments based on Duncan’s multiple range test.

NaCl stress severely limits the overall growth of rice seedlings, and foliar application of nano-silicon improves the water-holding capacity of leaves and induces coordinated above-ground and below-ground growth of rice seedlings. As shown in Figure 2, relative to CK, the S treatment significantly reduced the RWC of leaves by 14.44%, R/S by 15.26%, and seedling index by 34.35%. Compared to the S treatment, the SN treatment significantly increased the RWC of leaf by 6.09%, R/S by 11.06%, and seedling index by 24.83%.

### 2.4. Effects of Nano-Silicon on the Photosynthetic System of Rice Seedlings Under NaCl Stress

**Figure 3:**
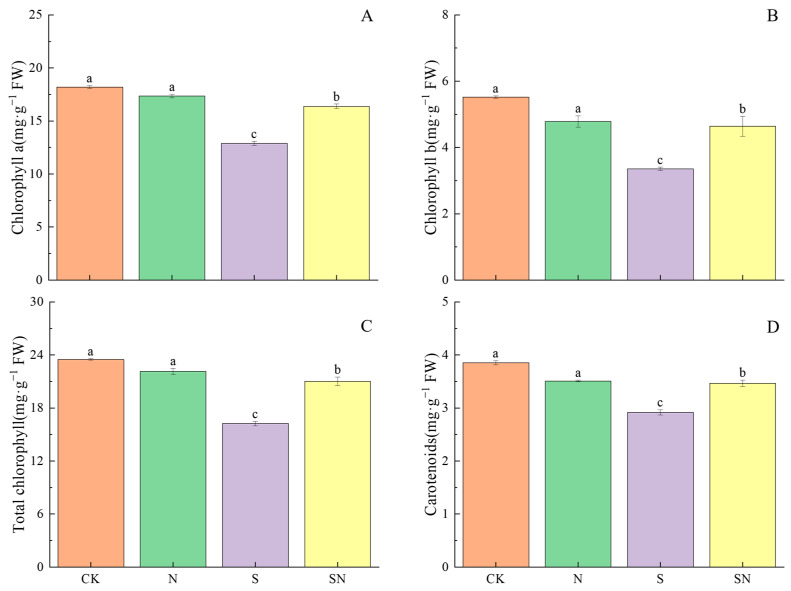
Effects of nano-silicon on photosynthetic pigment content in leaves of rice seedlings under NaCl stress. (Control (CK), nano-silica treatment (N), salt treatment (S), and nano-silica + salt treatment (SN). Chl a (**A**), Chl b (**B**), total chl (**C**), Car (**D**) in rice seeding). Different lowercase letters indicate significant difference at the 0.05 level among different treatments based on Duncan’s multiple range test.

NaCl stress resulted in reduced photosynthetic pigment content in rice seedling leaves, and foliar application of nano-silicon significantly alleviated photosynthetic pigment degradation due to NaCl stress. When compared to CK, the contents of Chl a, Chl b, total chl, and Car in the S treatment were all significantly reduced, by 29.19%, 39.12%, 30.84%, and 24.25%, respectively (Figure 3). In contrast to the S treatment, the SN treatment resulted in a significant increase in the content of Chl a, Chl b, total chl, and Car, with improvements of 27.14%, 38.17%, 29.42%, and 18.68%, respectively.

NaCl stress significantly inhibited photosynthesis in rice seedlings, and foliar application of nano-silicon improved the gas exchange parameters of leaves under NaCl stress and significantly enhanced photosynthesis in rice seedlings. As shown in Table 4, compared to CK, the S treatment resulted in a decrease in Pn, Tr, Ci, and Gs by 39.21%, 57.57%, 14.76%, and 72.70%, respectively. Compared to the S treatment, the SN treatment showed a significant increase in Pn, Tr, Ci, and Gs, with increases of 27.20%, 40.85%, 9.14%, and 90.00%, respectively. In comparison to CK, the S treatment led to a decrease in WUE and AMC by 31.48% and 20.35%, respectively, while the Ls increased by 58.45% compared to CK (Table 4). Compared to the S treatment, the SN treatment significantly increased WUE and AMC, by 27.86% and 9.89%, respectively, and significantly decreased Ls, by 7.87%.

### 2.5. Effects of Nano-Silicon on Membrane Damage Indices and Reactive Oxygen Species Accumulation in Rice Seedlings Under NaCl Stress

**Figure 4:**
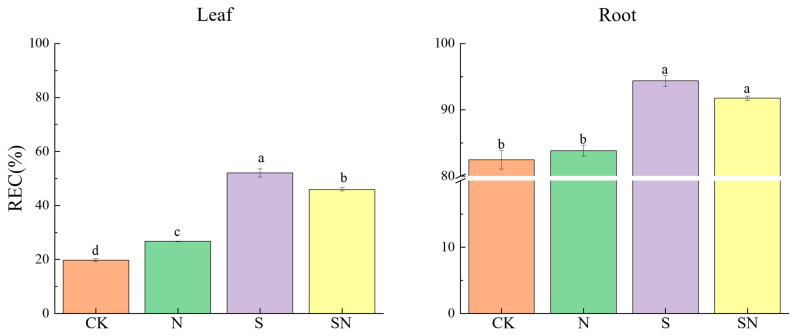
Effects of nano-silicon on REC in rice seedlings under NaCl stress. (Control (CK), nano-silica treatment (N), salt treatment (S), and nano-silica + salt treatment (SN)). Different lowercase letters indicate significant difference at the 0.05 level among different treatments based on Duncan’s multiple range test.

**Figure 5:**
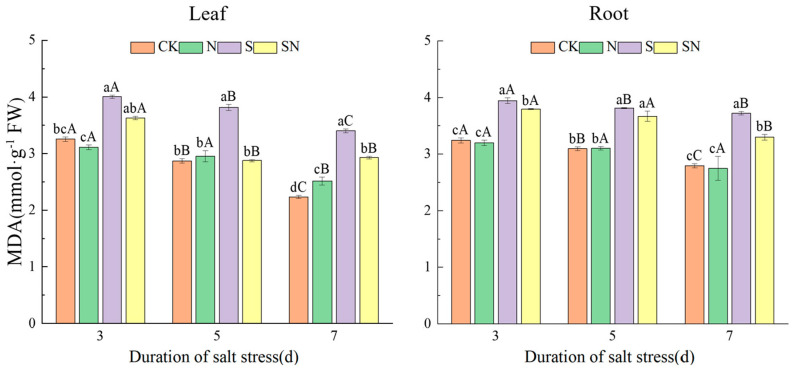
Effects of nano-silicon on MDA content in rice seedlings under NaCl stress. (Control (CK), nano-silica treatment (N), salt treatment (S), and nano-silica + salt treatment (SN). Note: 3, 5, 7 d indicates the duration of NaCl treatment. Lower case letters indicate differences between treatments at the same sampling time, upper case letters indicate differences between the same treatments at different sampling times (*p* < 0.05).

**Figure 6:**
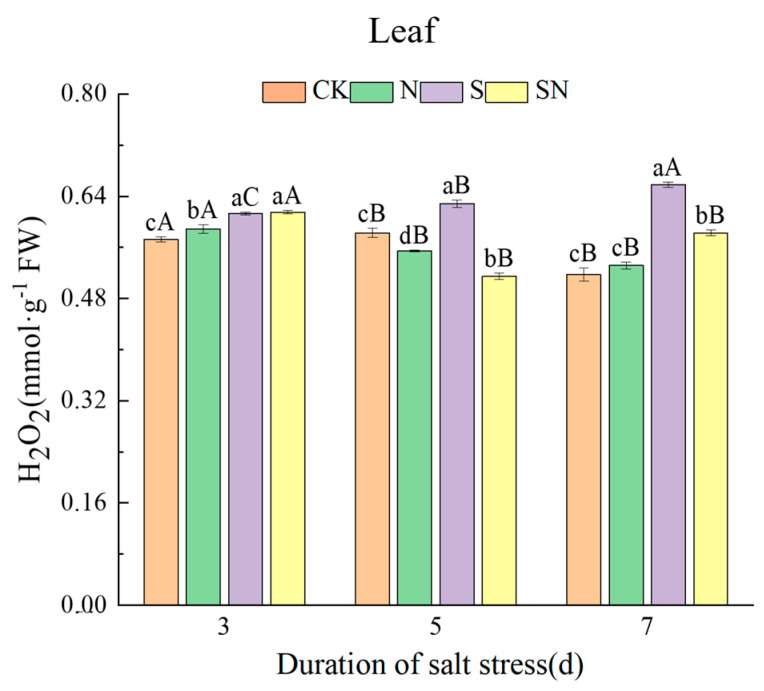
Effects of nano-silicon on H_2_O_2_ content in leaf of rice seedlings under NaCl stress. (Control (CK), nano-silica treatment (N), salt treatment (S), and nano-silica + salt treatment (SN)). Lower case letters indicate differences between treatments at the same sampling time, upper case letters indicate differences between the same treatments at different sampling times (*p* < 0.05).

NaCl stress caused rice seedlings to accumulate excessive ROS, resulting in cell membrane lipid peroxidation, leading to increased electrolyte extravasation. Foliar application of nano-silicon significantly reduced NaCl-induced ROS accumulation and attenuated cellular electrolyte extravasation. In comparison to CK, the S treatment significantly increased REC of leaves by 164.25% and roots by 14.41% (Figure 4). Compared to the S treatment, the SN treatment significantly reduced the leaf electrical conductivity, by 11.82%, while the change in root conductivity was not significant. As shown in Figure 5 and Figure 6, after 3, 5, and 7 d of NaCl treatment, there was a significant reduction in leaf and root MDA content in both the S and SN treatments. Under NaCl stress for 3 to 7 d, the S treatment increased MDA in leaves by 23.20% to 52.23% and in roots by 21.65% to 33.16%, respectively, when compared to CK. Compared to CK, the H_2_O_2_ content in leaves under the S treatment increased by 7.11% to 27.12%. When compared to the S treatment, the SN treatment significantly reduced the MDA content in leaves and roots by 9.43% to 24.62% and 3.74% to 11.37%, respectively. Additionally, after 3, 5, and 7 d of NaCl treatment, the H_2_O_2_ content of S-treated leaves increased with progressive time under stress, whereas the H_2_O_2_ content of the SN-treated leaves decreased significantly. Compared to the S treatment, the H_2_O_2_ content under the SN treatment was significantly lower, by 18.12% and 11.43%, on days 5 to 7 of stress.

To visualize the distribution of O_2_^·−^, histochemical staining was performed on the leaf of rice seedlings. O_2_^·−^ appeared as dark blue spots in the leaf (Figure S3). Compared to CK, the leaf of rice seedlings in the S treatment exhibited a significant increase in the number of dark blue spots, which were densely distributed. In contrast to the S treatment, the SN treatment resulted in a significant reduction in the number of dark blue spots on the leaf. The results showed that foliar application of nano-silicon effectively resisted the oxidative damage induced by NaCl stress.

### 2.6. Effects of Nano-Silicon on Antioxidant Enzymes in Rice Seedlings Under NaCl Stress

**Figure 7:**
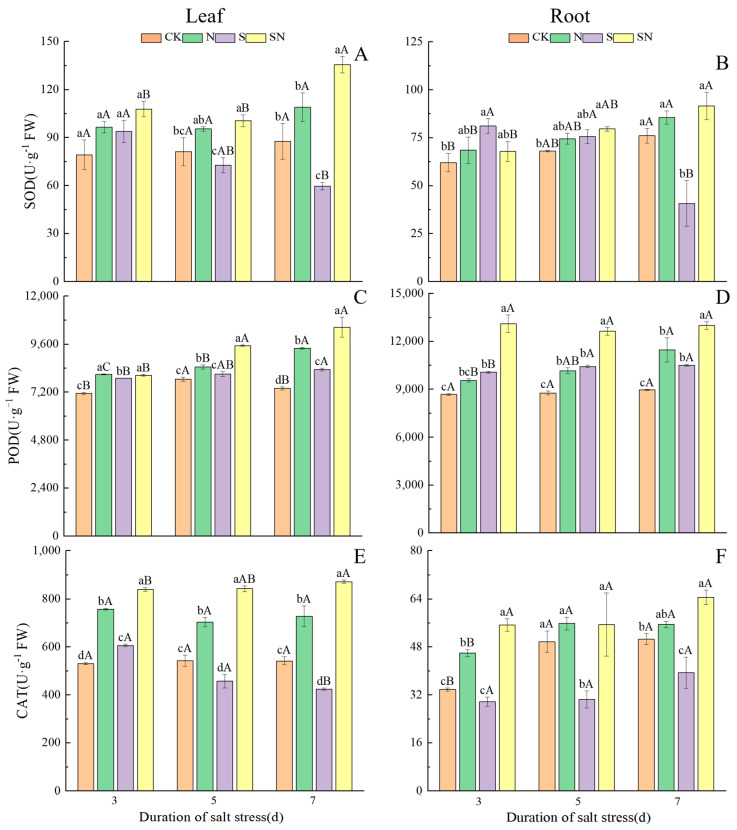
Of nano-silicon on antioxidant enzyme activities of rice seedlings under NaCl stress. (Control (CK), nano-silica treatment (N), salt treatment (S), and nano-silica + salt treatment (SN). SOD activity in leaf (**A**) and root (**B**), POD activity in leaf (**C**) and root (**D**), CAT activity in leaf (**E**) and root (**F**) of rice seeding). Lower case letters indicate differences between treatments at the same sampling time, upper case letters indicate differences between the same treatments at different sampling times (*p* < 0.05).

Figure 7 demonstrates the changes in the activities of antioxidant enzymes (SOD, POD, and CAT) in leaves and roots of rice seedlings under different treatments. After NaCl treatment, the SOD activities of both the leaves and roots of S-treated rice seedlings decreased with increasing time under stress, while the SOD activities of those undergoing SN treatment were significantly higher (Figure 7A,B). After 7 d of NaCl treatment, compared with CK, the SOD activities in the leaves and roots of S-treated rice seedlings were significantly reduced, by 31.98% and 46.49%, respectively. Compared with the S treatment, the SOD activities of the leaves and roots of SN-treated rice seedlings were significantly increased by 127.60% and 125.12%, respectively. After NaCl treatment, the POD activities of leaves and roots of both S-treated and SN-treated rice seedlings increased with increasing time under stress, but the elevated POD activities in the leaves and roots of SN-treated seedling were more significant (Figure 7C,D). After 7 d of NaCl treatment, compared to CK, the POD activities in S-treated leaves and roots increased by 12.80% and 17.14%, respectively. Compared to the S treatment, the POD activities of SN-treated leaves and roots were significantly increased by 25.39% and 23.66%, respectively. After NaCl treatment, CAT activity in the S-treated leaves decreased with increasing time under stress, and there was an increase in CAT activity in SN-treated leaves with increasing time (Figure 7E,F). After 7 d of NaCl treatment, compared with CK, the CAT activities of the S-treated leaves and roots were significantly reduced by 21.67% and 22.13%, respectively. Compared with the S treatment, the CAT activities of the leaves and roots undergoing the SN treatment were significantly increased by 105.47% and 63.79%, respectively. The results showed that foliar application of nano-silicon increased the antioxidant enzyme activity of rice seedlings and, at the same time, accelerated the response rate of antioxidant enzyme activity and enhanced the antioxidant capacity of the plants.

### 2.7. Effect of Nano-Silicon on Ascorbate-Glutathione Cycle in Rice Seedlings Under NaCl Stress

**Figure 8:**
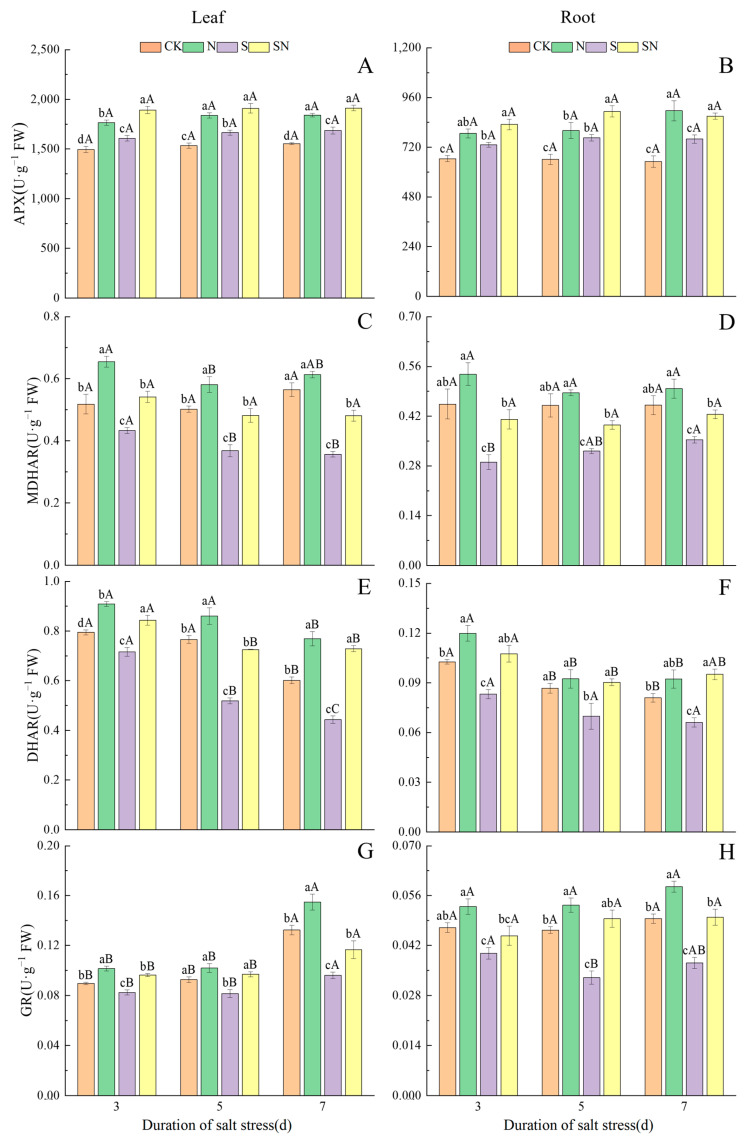
Effects of nano-silicon on the activities of AsA-GSH cycle-related enzymes of rice seedlings under NaCl stress. (Control (CK), nano-silica treatment (N), salt treatment (S), and nano-silica + salt treatment (SN). APX activity in leaf (**A**) and root (**B**), MDHAR activity in leaf (**C**) and root (**D**), DHAR activity in leaf (**E**) and root (**F**), GR activity in leaf (**G**) and root (**H**) of rice seeding). Lower case letters indicate differences between treatments at the same sampling time, upper case letters indicate differences between the same treatments at different sampling times (*p* < 0.05).

The AsA-GSH cycle-related enzyme activities of rice seedlings in each treatment can be seen in Figure 8, where NaCl stress increased APX activity in leaves and roots, but caused a decrease in MDHAR, DHAR, and GR activities. After NaCl treatment, APX activity in S-treated leaves and roots did not change with increasing time under stress, and APX activity in SN-treated leaves and roots was not affected by time under NaCl stress (Figure 8A,B). Compared with CK, APX activity was significantly elevated by 7.48–8.51% in S-treated leaves and 10.03–16.79% in roots at d 3, 5, and 7 after NaCl treatment. Compared to the S treatment, the APX activity was significantly higher in the SN treatment, by 13.36 to 17.75% in the leaves and 13.42 to 16.68% in the roots. After NaCl treatment, the MDHAR activity of S-treated leaves decreased with increasing time under stress, while the MDHAR activity of the root system increased, and SN-treated leaf and root MDHAR activities did not differ significantly across the three sampling occasions (Figure 8C,D). MDHAR activity in S-treated leaves was 16–36% lower than in CK at 3, 5, and 7 days after NaCl treatment. MDHAR activity in the root system was 21–36% lower. MDHAR activities of SN-treated leaves were higher by 24.98–34.68%, and activities in roots were higher by 20.29–41.46%, than those of the S treatment. After NaCl treatment, the DHAR activity of S-treated leaves significantly decreased with increasing time under stress, and a decrease in SN-treated leaf and root DHAR activity was also observed across the three sampling occasions (Figure 8E,F). Compared with CK, the DHAR activity of S-treated leaves was significantly reduced by 9.83 to 32.24%, while the DHAR activity of the root system was reduced by 18.40 to 19.38%, at 3, 5, and 7 days after S treatment. In comparison to the S treatment, the DHAR activity of SN-treated leaves was significantly increased, by 17.70 to 64.41%, and that of the root system was increased by 29.21 to 43.87%. After NaCl treatment, the GR activity of S-treated and SN-treated leaves showed an increasing trend at 3, 5, and 7 d after NaCl treatment, while the GR activity of S-treated roots gradually decreased, and there was no significant change in the GR activity of SN-treated roots (Figure 8G,H). At 3, 5, and 7 days after S treatment in comparison to CK, the GR activity of S-treated leaves was significantly reduced, by 8.03 to 27.36%, and that of the root system was decreased by 15.32 to 28.73%. Compared with the S treatment, the GR activity of SN-treated leaves was significantly increased by 16.73 to 21.44%, and that of the root system by 12.31 to 50.00%. The results showed that foliar application of nano-silicon enhanced the activities of enzymes related to the AsA-GSH cycle and helped to improve the antioxidant capacity of rice seedlings.

**Figure 9:**
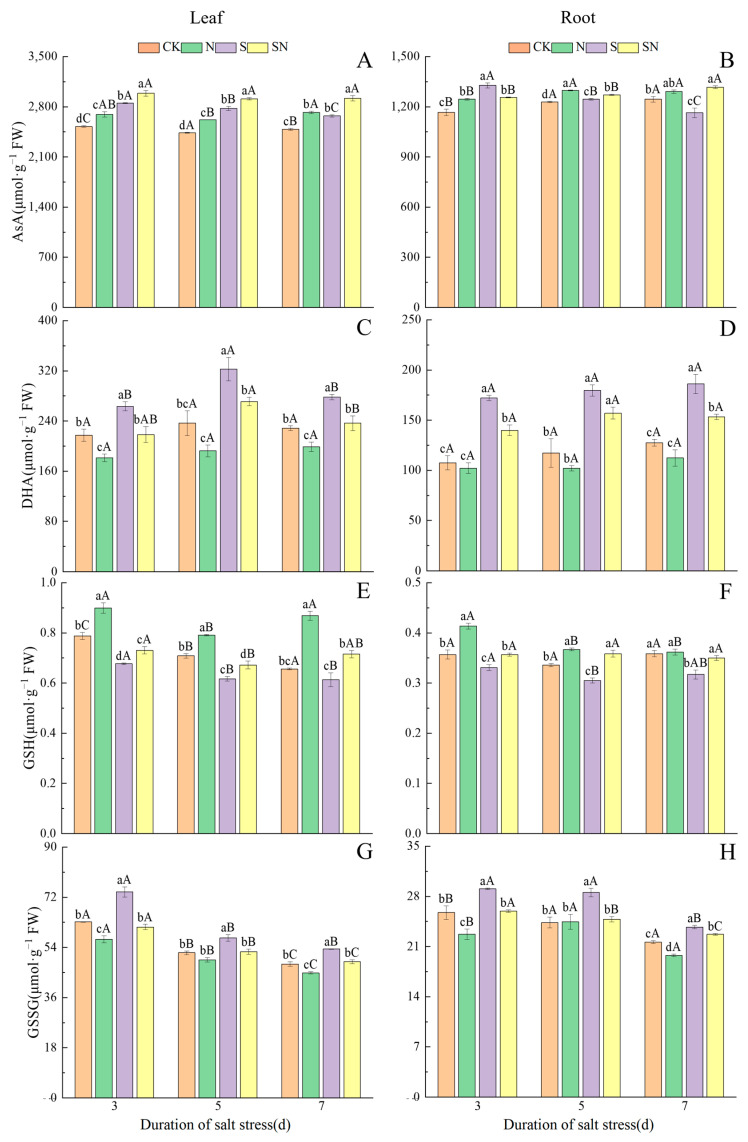
Effect of nano-silicon on the content of AsA-GSH cycle-related substances in rice seedlings under NaCl stress. (Control (CK), nano-silica treatment (N), salt treatment (S), and nano-silica + salt treatment (SN). AsA content in leaf (**A**) and root (**B**), DHA content in leaf (**C**) and root (**D**), GSH content in leaf (**E**) and root (**F**), GSSG content in leaf (**G**) and root (**H**) of rice seeding). Lower case letters indicate differences between treatments at the same sampling time, upper case letters indicate differences between the same treatments at different sampling times (*p* < 0.05).

**Figure 10:**
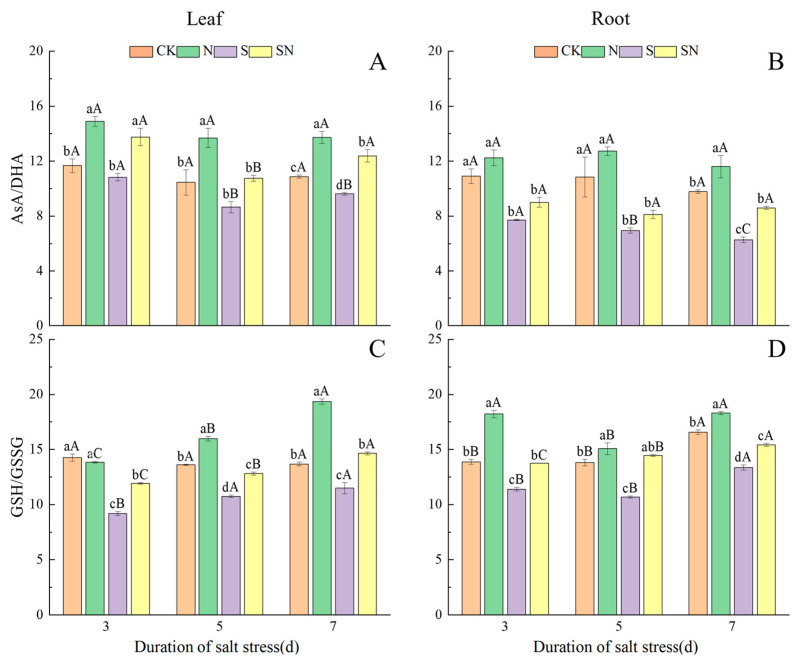
Effect of nano-silicon on AsA/DHA and GSH/GSSG ratios in rice seedlings under NaCl stress. (Control (CK), nano-silica treatment (N), salt treatment (S), and nano-silica + salt treatment (SN). AsA/DHA in leaf (**A**) and root (**B**), GSH/GSSG in leaf (**C**) and root (**D**) of rice seeding). Lower case letters indicate differences between treatments at the same sampling time, upper case letters indicate differences between the same treatments at different sampling times (*p* < 0.05).

Figure 9 demonstrates the changes in the contents of AsA-GSH cycle-related substances in rice seedlings at each sampling time under different treatments. NaCl stress significantly increased the contents of DHA and GSSG in the leaves and roots of rice seedlings, and increased the AsA content of leaves, but the AsA content of roots showed a tendency of increasing and then decreasing. After NaCl treatment, the AsA content of S-treated leaves and roots decreased significantly with increasing time under stress, and the AsA content of SN-treated leaves and roots was relatively stable. At 3, 5, and 7 days after the S treatment compared with CK, the AsA content of S-treated leaves was significantly elevated, by 6.71 to 7.58%, and that of the root system was reduced by 1.30 to 6.48%. In comparison to the S treatment, the AsA content of leaves in the SN treatment was significantly higher, by 9.16–10.88%, and the AsA content of the root system was significantly lower, by 3.51–13.83%. After undergoing NaCl stress, the DHA content of S-treated leaves increased and then decreased, while the DHA content of roots gradually increased but was not significantly different, and that of SN-treated leaves decreased significantly. At 3, 5, and 7 days after the S treatment compared to CK, the DHA content of S-treated leaves was significantly elevated, by 21.29 to 36.36%, and that of the root system was significantly reduced, by 45.99 to 59.99%. In comparison to the S treatment, DHA content was significantly reduced, by 14.89 to 17.14% in SN-treated leaves, and significantly elevated, by 12.57 to 18.75%, in the root system. As a result, the AsA/DHA rate of S-treated leaves and roots significantly decreased at 3, 5, and 7 d of NaCl stress, while the AsA/DHA rate of SN-treated leaves decreased and then increased, and there was no significant change in the AsA/DHA rate of roots. Compared to CK, AsA/DHA was significantly reduced, by 11.57 to 21.16% in S-treated leaves and 35.58 to 41.41% in the root system. AsA/DHA was significantly increased, by 28.26 to 33.82% in leaves and 18.39 to 40.10% in roots of the SN treatment in comparison to the S treatment (Figure 10). After 3, 5, and 7 d of NaCl treatment, the GSH content of S-treated leaves and roots gradually decreased, while the GSH content of SN-treated leaves and roots was relatively stable but not significantly different. At 3, 5, and 7 days after NaCl treatment, in comparison to CK, the GSH content of S-treated leaves was significantly reduced, by 6.57 to 14.01%, while that of the root system was reduced by 7.26 to 11.56%. Compared to the S treatment, the GSH content of SN-treated leaves was significantly increased, by 7.89 to 16.57%, and that of the root system was significantly increased, by 7.83 to 17.51%. The GSSG content of S-treated leaves and roots gradually decreased at 3, 5, and 7 d after NaCl treatment, and the GSSG content of SN-treated leaves and roots significantly decreased with increasing time under stress. Compared with CK, the GSSG content of S-treated leaves was significantly increased, by 10.22 to 17.02%, and that of the root system was increased by 9.76 to 17.21% at 3, 5, and 7 days after the S treatment. In comparison to the S treatment, the GSSG content was significantly reduced, by 8.51 to 16.99% in the leaves and 4.30 to 13.07% in the root system of the SN treatment. As a result, GSH/GSSG gradually increased in S-treated leaves and roots at 3, 5, and 7 d of NaCl stress, and significantly increased in SN-treated leaves and roots with increasing time of nano-silicon treatment. Compared to CK, GSH/GSSG was significantly reduced, by 16.02 to 35.65% in S-treated leaves and 17.95 to 22.65% in the root system. Compared to the S treatment, GSH/GSSG was significantly increased by 19.16 to 29.88% in leaves and 15.36 to 35.17% in the root system in the SN treatment. The results showed that foliar application of nano-silicon could regulate the AsA/DHA and GSH/GSSG ratios in leaves and roots of rice seedlings under NaCl stress to enhance the antioxidant capacity of the plants.

### 2.8. Effects of Nano-Silicon on the Pro and SP Contents in Rice Seedlings Under NaCl Stress

**Figure 11:**
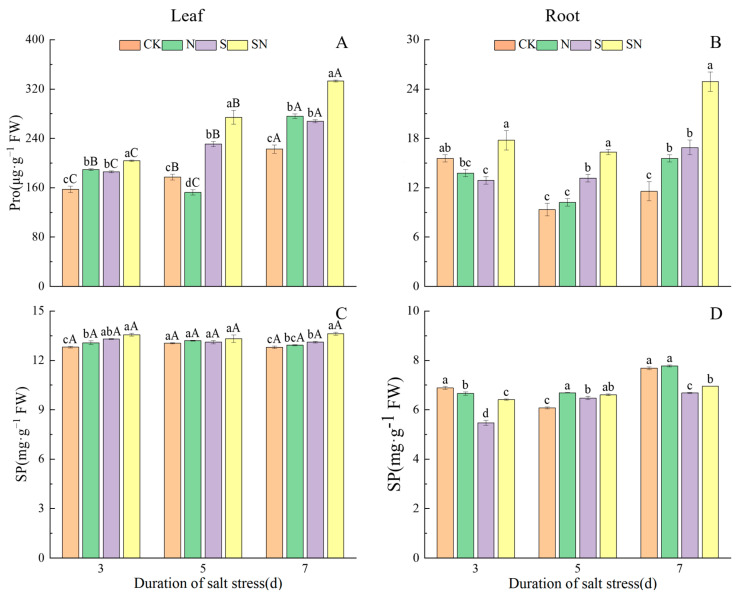
Effects of nano-silicon on soluble protein and proline contents in rice seedlings under NaCl stress. (Control (CK), nano-silica treatment (N), salt treatment (S), and nano-silica + salt treatment (SN). Pro content in leaf (**A**) and root (**B**), SP content in leaf (**C**) and root (**D**) of rice seeding). Lower case letters indicate differences between treatments at the same sampling time, upper case letters indicate differences between the same treatments at different sampling times (*p* < 0.05).

NaCl stress caused the accumulation of osmoregulatory substances in the leaves and roots of rice seedlings, and the Pro content was higher than that of CK in both S-treated leaves and roots, while the SP content of S-treated leaves was also higher than that of CK, but the SP content of roots was significantly lower. After 3, 5, and 7 d of NaCl treatment, the Pro content of leaves and roots under both S and SN treatments increased significantly with the increase in time under stress, and there was no significant difference between the SP content of leaves in the S and SN treatments. In comparison to CK, the SP and Pro contents in the leaves of the S treatment group were significantly increased, by 24.07% and 20.17%, respectively. Furthermore, the Pro content in the roots of the S treatment showed a significant increase, of 47.37%, while the SP content was significantly reduced, by 13.02% (Figure 11). As opposed to the S treatment, the SN treatment led to notably higher levels of both SP and Pro in the leaf and root systems, with respective augmentations of 3.95%, 4.04%, 24.42%, and 47.37%. The results showed that foliar application of nano-silicon increased Pro and SP contents in leaves and roots of rice seedlings and improved osmotic potential to withstand NaCl stress.

### 2.9. Effects of Nano-Silicon on Growth Hormone Content of Rice Seedlings Under NaCl Stress

As shown in Table 5, S stress significantly affected the endogenous hormone levels of each plant, and foliar application of nano-silicon could effectively reverse the effects of NaCl. In comparison to CK, the S-treated leaves showed a significant reduction of 34.70% in IAA, 28.51% in CTK, 34.97% in GA, 47.11% in ABA, 18.08% in SA, and 25.75% in JA, and the root system showed a significant reduction of 32.61% in IAA, 38.89% in CTK, 17.00% in GA, while ABA significantly increased by 30.69%, SA significantly increased by 27.73%, and JA significantly increased by 9.14%. In comparison to the S treatment, the SN treatment showed a significant increase of 22.08% in IAA, 30.87% in CTK, 36.70% in GA, 15.45% in ABA, 32.17% in SA, and 22.21% in JA in the leaves, and in the root system it showed a significant increase in IAA by 56.14%, GA by 2.65%, ABA by 13.23%, SA by 27.71%, and JA by 24.62%.

### 2.10. Effect of Nano-Silicon on Ion Content of Rice Seedlings Under NaCl Stress

The ion content of seedling leaves and roots systems varied significantly and differently under different treatments (Table 6). Compared to CK, Na^+^ was significantly elevated by 133.20% and 419.06%, in the leaves and roots of the S treatment. Compared to the S treatment, Na^+^ was significantly reduced, by 13.29% and 7.12%, in the leaves and roots of the SN treatment, respectively. Compared to CK, Cl^−^ was significantly elevated, by 241.34% and 165.69%, in the leaves and roots of the S treatment, respectively. Compared to the S treatment, Cl^−^ was significantly reduced by 23.24% and 4.04% in the leaves and roots of the SN treatment, respectively. Compared to CK, Si^4+^ was significantly reduced by 33.98% and 23.17% in S-treated leaves and roots, respectively. Compared to the S treatment, Si^4+^ was significantly elevated, by 73.84% and 59.28%, in leaves and roots of the SN treatment, respectively. Compared to CK, K^+^ was significantly reduced, by 14.06% and 41.30%, in S-treated leaves and roots, respectively. Compared to the S treatment, the K^+^ of leaves and roots of the SN treatment was significantly higher, by 6.89% and 9.15%, respectively. Compared to CK, Ca^2+^ was significantly reduced, by 5.49% and 47.12%, in the leaves and roots of the S treatment, respectively. Compared to the S treatment, Ca^2+^ was significantly higher in the leaves and roots of the SN treatment, by 7.91% and 82.20%, respectively. As a result, in comparison to CK, Na^+^/K^+^ was significantly reduced, by 181.00% and 785.11%, in S-treated leaves and roots, respectively. Compared to the S treatment, Na^+^/K^+^ of leaves and roots in the SN treatment was significantly increased, by 18.89% and 14.94%, respectively. The results showed that foliar application of nano-silicon could reverse the ionic imbalance caused by NaCl stress, reduce Na^+^ and Cl^−^ uptake, and promote K^+^ and Ca^2+^ uptake.

### 2.11. Changes in Stomata and Chloroplast Ultrastructure of Rice Seedling Leaves

Scanning electron microscopy observations revealed significant changes in the number of trichomes (Tr) and the degree of stomatal closure in rice seedling leaves after foliar application of nano-silicon. The number of Tr on the leaf surface of rice seedlings was significantly increased in the SN treatment compared to the S treatment (Figure S4C,D). Meanwhile, the stomata of rice leaves were almost entirely closed in the S treatment and closed significantly in the SN treatment compared to CK (Figure S4E,G,H). Transmission electron microscopy observations showed that there was a large amount of flocculent material in the leaf cells, impaired structural integrity of chloroplasts, ruptured chloroplast periplasm, and dried and thin starch grains in the S treatment (Figure S5C) compared to CK (Figure S5A). As shown in Figure S5D, in comparison to the S treatment, there was less flocculent in the leaf cells, chloroplasts were significantly enlarged and structurally intact, and starch granules were full and rounded in the SN treatment in rice.

### 2.12. Two-Factor Analysis and Correlation Analysis

Data on MDA, H_2_O_2_, osmoregulatory substances, and antioxidant enzymes in Table S1 showed that the treatments had significant effects on MDA, H_2_O_2_, Pro, SP, SOD, POD, CAT, APX, MDHAR, DHAR, and GR at the sampling times. The results indicated that both NaCl stress and foliar application of nano-silicon had a more stable stress/mitigation effect on rice seedlings throughout the treatments.

The experiment screened rice seedlings for morphological growth, photosynthetic gas exchange parameters, endogenous plant hormones, ion content, and AsA-GSH cycle-associated antioxidant enzyme activities with non-enzymatic antioxidants for correlation analysis (Figure S6). The results showed that IAA and GA were significantly and positively correlated with the biomass, seedling index, root spatial configuration, and photosynthetic gas exchange parameters of rice seedlings, and CTK content was significantly and positively correlated with root spatial configuration-related parameters; moreover, the increase in IAA, CTK and GA content could reduce the accumulation of Na+ in plants, and also reduce the level of the ABA hormone to break its morphological growth and photosynthesis inhibitory effects. Meanwhile, it was found that the contents of DHA and GSSG in the AsA-GSH cycle were all significantly negatively correlated with the morphological growth parameters of rice seedlings, while the activities of MDHAR and GR were inversely correlated, and there was a significant positive correlation between the activities of MDHAR and GR and the levels of CTK and GA hormones, respectively.

## 3. Discussion

### 3.1. Effect of Nano-Silicon on Morphological Growth Parameters of Rice Seedlings Under NaCl Stress

Soil salinization is a factor affecting food security due to its persistence and toxicity in environmental systems [28]. In general, salt stress, primarily NaCl stress, inhibits plant growth and development [29], and NaCl concentrations above the tolerance limit have severe negative effects on crop production. Furthermore, high Na^+^ concentrations in the growth environment can cause cellular osmotic pressure imbalances, leading to water uptake difficulties, and can also cause excessive uptake of intracellular salt ions, which can lead to oxidative stress in cells. This results in the denaturation of cell membrane lipids and the disruption of normal cellular organelle functioning, ultimately manifesting as abnormal cell growth or even death [30]. Studies have reported [31] that salt stress reduces plant growth, leaf area index, and photosynthetic pigment content, while also leading to the greening of leaf in rice. In this study, we found that NaCl stress significantly inhibited the normal above- and below-ground growth of rice seedlings, which manifested as a decrease in growth indices such as plant height, leaf area, root length, and biomass. This, in turn, significantly reduced the relative water content of leaves, root crown ratio, and firmness index; furthermore, NaCl stress limited the spatial conformation in the root systems of seedlings. Numerous studies [32] have shown that silica nanomaterials can significantly increase the stress tolerance of plants and improve the growth status of plants under NaCl stress. The foliar application of 2.00 mmol·L^−1^ nano-silicon significantly increased the biomass of rice seedlings subjected to saline stress, and their root system spatial configuration was significantly improved, with better promotion of the total root length, root volume, root thickness, and number of root forks and apices (Table 3). Yetgin et al. reported that the promotion of root elongation is crucial for enhancing a plant’s ability to absorb water and nutrients, thereby improving overall growth conditions [33]. The total chlorophyll and carotenoid contents of NaCl-treated plants were significantly reduced compared to the control, and the application of nano-silicon significantly increased the net photosynthetic rate, transpiration rate, and stomatal conductance of rice seedlings under NaCl stress treatment (Table 4), which was similar to the results of a previous study [34]. Furthermore, the study revealed that NaCl stress not only resulted in stomatal closure in rice seedling leaves (Figures S4 and S5), but also led to damage to chloroplasts and disruption of cellular structure; the foliar application of nano-silicon maintained the stomatal opening of the leaf, effectively protected the integrity of chloroplasts, and promoted starch accumulation (Figure S5). Correlation analyses revealed that the spatial configuration of the root system of rice seedlings was significantly positively correlated with the gas exchange parameters of the leaf, and that the protection and enhancement of photosynthesis of the leaf could provide sufficient energy for the growth of the root system [35]. Furthermore, coordinated root growth could provide sufficient water and nutrient elements for the leaf [33]. Therefore, we concluded that the application of exogenous nano-silicon can promote the growth of rice seedlings under NaCl stress, coordinate the growth of both the above-ground and underground systems, and to a certain extent maintain their normal photosynthesis, which has a significant mitigating effect on salt damage.

### 3.2. Effect of Nano-Silicon on Antioxidant System and Reactive Oxygen Species Accumulation in Rice Seedlings Under NaCl Stress

Salt stress causes irreversible damage to the electron transfer process in plants, and this damage generates a large amount of reactive oxygen species (ROS), which, if not scavenged in time, can accumulate in excess, causing cell membrane lipid peroxidation and disrupting normal physiological and metabolic processes in plants [8]. When the crop growth process is subjected to salt stress, effective antioxidant defense systems, such as antioxidant enzymes, can play an important role in scavenging ROS and enhancing plant antioxidant activity [36]. In this study, the antioxidant enzyme activities of seedlings exhibited different performances with increases in time under NaCl stress, and the SOD and CAT activities of NaCl-treated leaves first increased and then decreased compared with the control treatment, which was assumed to be the up-regulation of enzyme activities stimulated by NaCl stress; however, as the exposure time increased, the accumulation of ROS was too high and ROS could not be efficiently scavenged in time, causing oxidative damage to the cells [9]. This is similar to the trend of antioxidant enzyme changes in rice under NaCl stress found in a previous study [37]. After the foliar application of nano-silicon, it was found that nano-silicon alleviated the adverse effects of NaCl stress on rice seedlings by promoting the increase in the activities of antioxidant enzymes (SOD, POD, and CAT) and scavenging the excess ROS (H_2_O_2_, O_2_^·−^), thus reducing the degree of membrane damage (MDA) in seedlings. Badawy et al. also found that nano-silicon and nano-zinc mitigated the negative effects of NaCl stress by increasing the antioxidant enzyme activity and biomass of rice [22]. Riaz indicated that nano-silicon mitigated the toxicity of Cd to rice by enhancing antioxidant defenses [23]. Faten observed that the electrolyte permeability of the plant was significantly increased under NaCl stress conditions and membrane damage intensified, whereas exogenous nano-silicon could enhance the antioxidant capacity of the crop by increasing the antioxidant enzyme activity and promoting plant growth [38].

The antioxidant defense mechanism in higher plants consists of antioxidant enzymes and non-enzymatic antioxidants, of which the ascorbate–glutathione cycle is an important component, with AsA and GSH being key non-enzymatic constituents. The AsA-GSH cycle is able to effectively hydrolyze the H_2_O_2_ produced by physiological metabolic processes and plays a key role in the scavenging of ROS [39]. AsA, also known as vitamin C, is an electron donor in redox reactions and is able to mitigate salt stress damage to plant cells. The involvement of APX, DHAR, and MDHAR in the oxidation and synthesis cycle of AsA improves the antioxidant capacity of plants [40]. In this study, NaCl stress caused an increase in the overall AsA content of rice seedlings, suggesting that the synthesis of AsA was stimulated after the plants were subjected to stress, followed by the production of DHA from AsA catalyzed by APX, a process that contributes to H_2_O_2_ scavenging. This can be verified from the results where an increase in the activity of APX was observed (Figure 8), in conjunction with an increase in the DHA content of the seedlings (Figure 9). After foliar application of nano-silicon, we found that the APX activity and AsA content were further increased and the DHA content was decreased compared with NaCl treatment, accompanied by the enhancement of DHAR activity and MDHAR activity, and the reduction in MDA and ROS (H_2_O_2_, O_2_^·−^), suggesting that nano-silicon could further enhance the antioxidant enzyme activity and accelerate the oxidative reduction of AsA as a means to reduce the excessive ROS accumulation brought about by oxidative stress [11]. The AsA/DHA ratio is an important indicator of antioxidant status; as shown in Figure 10, rice seedlings after foliar application of nano-silicon had a higher AsA/DHA ratio than in NaCl treatment, suggesting that nano-silicon treatment was able to maintain higher levels of AsA in the reduced state, which helped to maintain strong antioxidant capacity, as reported by Fan et al. [41].

GSH, as an important non-enzymatic antioxidant in the AsA-GSH cycle, is oxidized by DHAR catalyzing the oxidation of GSH to produce GSSG and the scavenging of H_2_O_2_, followed by GR-mediated reduction to GSH to complete the cycle; the rate at which the cycle is completed greatly influences the potency of AsA [42]. The content and ratio of GSH and GSSG are other important indicators of cellular antioxidant status. In this study, the GSH content of rice seedlings significantly decreased after being subjected to NaCl stress (Figure 9), presumably in order to scavenge the large amount of accumulated ROS as soon as possible; however, we observed that the GR activity was also inhibited by NaCl stress, resulting in elevated GSSG content, which could not be reduced to GSH in a timely manner, and the rate of redox cycling was restricted, resulting in an excessive accumulation of ROS and causing damage to the cell membrane [43] (the MDA content was elevated). The GSH/GSSG ratio is an important indicator of plant antioxidant capacity, and after the foliar application of nano-silicon, we found that the GSG/GSSG ratio of rice seedlings increased significantly compared with NaCl treatment alone, and enhanced GR and DHAR activities could also be observed, which provided a sufficient pool of GSH for the plant to fight against the accumulation of ROS. This is consistent with a previous study [39], which found that nano-silicon alleviates oilseeds’ oxidative damage. Correlation analyses showed that the oxidation state of the AsA-GSH cycle was significantly negatively correlated with the growth of rice seedlings (Figure S6), while the Si^4+^ content was positively correlated with the GSH content and the GSH/GSSG ratio, and that increasing the level of the reduced state of the AsA-GSH cycle significantly improved the root conformation and leaf photosynthesis of rice seedlings. Therefore, we believe that the foliar application of nano-silicon can effectively enhance the activity of enzymes related to the AsA-GSH cycle, accelerate the redox cycle of the antioxidant substances AsA and GSH, and improve the antioxidant capacity of rice seedlings more comprehensively, to counteract the growth inhibitory effects of NaCl stress.

### 3.3. Effect of Nano-Silicon on the Accumulation of Osmoregulatory Substances in Rice Seedlings Under NaCl Stress

The osmotic stress experienced by plants during growth can be alleviated by increasing osmoregulatory substances in the plant to balance the cytoplasmic and external water potentials [44]. SP and Pro serve as key osmolytes; SP, being integral to cellular composition, can initiate responses to external stress and engage in osmotic adjustment, while Pro, acting as an osmoprotectant, is essential for plant recovery from environmental stressors [26]. In this study, it was found that the SP content of rice leaves increased under NaCl stress (Figure 11C), and the SP content of roots first increased and then decreased (Figure 11D). This may be due to the protective mechanism of rice in the early stage of NaCl stress, which caused the protein content to increase first; the content of SP then decreased in the later stages owing to the degradation of proteins under salt stress. NaCl stress in rice seedlings yielded an increasing trend in Pro content, indicating that plants increased the accumulation of osmoregulatory substances to adapt to the osmotic environment. Research has indicated that salt stress can lead to a suppression in protein synthesis or an acceleration in protein degradation [45], as the breakdown of SP can generate a substantial amount of free amino acids. Pro is among the first amino acids to rapidly increase in a variety of crops when exposed to stress [46]. In this study, the application of nano-silicon via foliar spray enhanced the levels of SP and Pro in both the leaves and roots of rice seedlings (refer to Figure 11). This enhancement helps maintain a lower osmotic potential within plant cells; increases the leaf relative water content, as shown in Figure 2A; and supports the normal development of plants even under the stress of saline conditions. In summary, nano-silicon has the capacity to modulate the water balance in rice seedlings and preserve leaf integrity by stimulating the accumulation of osmoprotectants, thereby alleviating the negative impacts of salt stress, a result consistent with Yan’s research [47].

### 3.4. Effect of Nano-Silicon on Endogenous Hormones in Rice Seedlings Under NaCl Stress

Endogenous hormones play a direct or indirect regulatory role in plant development, and plant responses to environmental changes are often reflected in changes in the levels of many endogenous hormones; when subjected to external stress, the dynamic balance of hormones in the plant is disrupted, including synthesis and catabolism [48]. In this experiment, after treatment with foliar application of nano-silicon, the endogenous hormone levels of rice seedling leaves and roots changed significantly, with IAA, CTK, GA, SA, and JA contents significantly increasing and ABA contents decreasing. Nanomaterials can mitigate the adverse effects of salt stress by regulating hormone levels (ABA, CTK) and affecting cell division and root growth [49]. Yadav et al. found that plants decrease ABA when they increase the levels of GA and CTK [50]. Weria et al. found that nano-silicon can activate signaling pathways mediated by SA and JA, which modulate the plant’s stress response mechanism [21]. Tripathi et al. suggested that plants increase JA levels under stress conditions and that JA participates in silica-regulated membrane signaling pathways [51]. In this experiment, endogenous hormone changes were significant in the leaves and roots of rice seedlings under NaCl stress, with significant increases in ABA, SA, and JA contents and decreases in the IAA, CTK, and GA contents. In comparison to salt stress, the endogenous hormone contents of seedlings changed significantly after the foliar application of nano-silicon, with a significant decrease in ABA content; a significant increase in IAA, CTK, and GA (Table 5); and a further increase in SA and JA content. Abouelsaad found that JA could improve plant salt tolerance by maintaining ROS homeostasis [52], and Wang reported that exogenous nano-silicon could enhance the expression of SA- and JA-related genes and increase the starting hormone levels to regulate the growth and antioxidant capacity of rice [53]. Correlation analysis determined that root conformation-related indexes showed a significant positive correlation with IAA and CTK (Figure S6), and a significant negative correlation with ABA. IAA and GA also showed a significant positive correlation with photosynthetic gas exchange parameters, and SA showed a positive correlation with antioxidant enzymes of the AsA-GSH cycle. This indicated that foliar application of nano-silicon modulated the level of endogenous hormones, and then each of the hormones acted to improve the root system by promoting its coordinated growth, which fed back to the above-ground part to improve the stoutness index of rice seedlings (Figure 2), guaranteeing the normal photosynthesis of leaves and increasing the overall biomass accumulation of the plant [54]. Therefore, we believe that the foliar application of nano-silicon can regulate the hormone levels of rice seedlings to improve salt tolerance and maintain normal physiological and biochemical functions.

### 3.5. Foliar Application of Nano-Silicon Modulates Ionic Homeostasis in Rice Seedlings

The dynamic balance of sodium and potassium ions is crucial for plant growth under salt stress, and high external concentrations of salt ions (e.g., Na^+^ and Cl^−^) can lead to physiological plant water deficits through osmotic stress; the root system passively accumulates a large amount of Na^+^ and Cl^−^, which hinders nutrient uptake and induces stress injuries, such as membrane lipid peroxidation and the disruption of the photosynthetic system [31]. Salt stress inhibits plant growth and development, and biomass accumulation is commonly used to characterize the severity of salt damage; it is a direct indicator of plant salt tolerance [55]. In this study, the large-scale accumulation of Na^+^ and Cl^−^ in NaCl-treated rice seedlings hindered the uptake and utilization of nutrients such as Si^4+^, K^+^, and Ca^2+^, causing nutrient imbalance in the plants and resulting in significant growth inhibition (Table 1 and Table 2). The foliar application of nano-silicon yielded a significant reduction in the Na^+^ and Cl^−^ contents of the seedlings, and a significant increase in the contents of Si^4+^ and Ca^2+^, which effectively alleviated the ion toxicity produced by NaCl stress (Table 6). Si^4+^ promotes participation in phenolic accumulation, enhances plant antioxidant capacity, and is also a well-known beneficial element for growth [56]. Ca^2+^, as an intracellular second messenger, not only interlinks with plant sensing in the face of salt stress, but also ensures subsequent signal transduction [57]. Previous studies have shown that rice can maintain the sodium–potassium balance by regulating K^+^ uptake, Na^+^ efflux, and compartmentalization [58]. In this study, we found that NaCl stress significantly reduced the K^+^ content of rice seedlings, and the foliar application of nano-silicon improved this but did not make a significant difference; rather, it served to reduce Na^+^/K^+^ by controlling the accumulation of Na^+^ uptake. In addition, after the foliar application of nano-silicon, the Na^+^ content of leaves decreased more than that of roots, and in combination with its promotional effect on the spatial conformation of roots (Table 3 and Figure S2), we speculate that nano-silicon is more biased in regulating Na^+^ uptake transporter-related genes, effectively reducing the accumulation of Na^+^ while potentially restricting its translocation to the above-ground region. Bosnic showed that exogenous silicon was able to increase the exocytosis of Na^+^ from the below-ground part of rice by increasing the expression of the Na^+^ exocytosis gene, *OsSOS1*, under salt stress conditions, thus decreasing its translocation to the above-ground part [59]. We also noted that the application of nano-silicon elevated the content of JA, IAA, and CTK in the root system higher than that in the leaves, which contributed to the growth and development of the root system, accumulating more Na^+^ while maintaining a certain level of Na^+^/K^+^. Gong reported a similar role of silicon in reducing the uptake and transport of Na^+^ [60]. In this study, we found that Na^+^, Cl^−^, and Na^+^/K^+^ were all significantly positively correlated with ABA (Figure S6), and significantly negatively correlated with GA and IAA, whereas the contents of Si^4+^ and Ca^2+^ were significantly positively correlated with CTK. An increase in the content of Si^4+^ also enhanced the reduced state of the GSH cycle and encouraged a greater antioxidant capacity in the rice seedlings, improving NaCl and ultimately improving the growth status of plants under NaCl stress.

## 4. Materials and Methods

### 4.1. Plant Material and Growth Conditions

The rice variety used in this experiment was 9311 (Yangdao 6, National Approval No. 2001002), a salt-sensitive cultivar, which was bred by the Jiangsu Lixiahe Regional Agricultural Science Institute. The seeds were chosen based on their fullness and disease-free status, then sanitized in a 3.00% (*v*/*v*) hydrogen peroxide solution for a duration of 15 min, and subsequently washed extensively with deionized water. They were then transferred into beakers containing distilled water and soaked under dark conditions for 24 h. Subsequently, the seeds were evenly spread on double-layer grid germination trays (32.00 cm × 25.50 cm × 12.00 cm) and placed in a 30 °C constant-temperature incubator (JIDI-PX1100, JIDI, Shenzhen, China) for germination under dark conditions; they were misted with ultrapure water once in the morning and evening to maintain seed moisture. Once the rice germ had reached a length of approximately 1.00 cm, it was transferred to a black hydroponic box containing 900 mL of distilled water and allowed to acclimatize for 12 h (48 plants per box). Subsequently, the water was replaced with half-strength Hoagland nutrient solution (pH 6.70 to 6.90) [61], and the seedlings were put in a growth chamber (with a photosynthetic photon flux density of 30,000 lux, a photoperiod of 14 h light/10 h dark, and temperatures of 28 °C/23 °C day/night) for cultivation, with the nutrient solution being renewed every 3 d.

### 4.2. Characterization of Nano-Silica Materials

The original concentration of the nano-silica material used in the experiment was 2.00 mol·L^−1^ (purchased from Beijing Suo Laibao Science & Technology Co., Ltd., Beijing, China). It was diluted with deionized water and dispersed in an ultrasonic bath for 30 min before further processing. Transmission electron microscopy (TEM, HT7700 Exalens, Hitachi, Tokyo, Japan) was used to observe the morphology of the nano-silica (Figure S7).

### 4.3. Experimental Design

The experiment was conducted from September 2022 to July 2024 at the South China Center for National Salt-Tolerant Rice Technology Innovation, Institute of Agricultural Biotechnology, Guangdong Ocean University.

Once the seedlings reached the 3-leaf and 1-heart stage, the foliar application of nano-silica solution was administered (10.00 mL per box at a concentration of 2.00 mmol·L^−1^, equivalent to an actual silica application rate of 1.20 mg per box, The concentrations used in the experiment were derived from the results of the previous concentration screening test (Table S2, with the optimal concentration of 2.00 mmol·L^−1^ in bold), with deionized water used as a control (the front and back sides of leaves were sprayed with a high-pressure spray bottle until wet, but not dripping). Twenty-four hours post-treatment, NaCl was added to simulate salt stress, achieving a final concentration of 60.00 mmol·L^−1^. Four treatments were established: control (CK), nano-silica treatment (N), salt treatment (S), and nano-silica + salt treatment (SN), with nine replicates for each treatment. The experimental design is schematically illustrated in Figure S8. Samples were collected at 3, 5, and 7 d post-salt treatment, flash-frozen with liquid nitrogen, and then transferred to a −40 °C freezer for storage and subsequent analysis. Each morphological indicator was measured 7 d after NaCl treatment.

### 4.4. Determination of Indices and Methods

#### 4.4.1. Morphological Indicators

In each replicate of each treatment, 20 uniform seedlings were randomly selected. Seedlings were divided into the shoot and root at the base of the stem, and their fresh weights (FWs) were measured separately. The leaves were removed from the leaf sheath, soaked in ultrapure water for 24 h, then quickly blotted dry with absorbent paper to measure the weight at full saturation (TW). After killing green matter at 105 °C for 30 min, they were dried to a constant weight at 80 °C, and the DWs of the leaves, stems, and roots were measured. The root systems of the seedlings were scanned using a desktop scanner (Epson CORP, Nagano, Japan), and the total root length, total root surface area, total root volume, and root tip numbers were obtained using Win RHIZO root analysis software (DJ-GX02, Regent Instruments, Inc., Quebec, QC, Canada). The root-to-shoot ratio (R/S), seedling index, and relative water content (RWC) [62] were calculated using the following formulas:

R/S = DW of root/shoot(1)

Seedling index = (R/S + Stem base width/Plant height) × (DW of root + shoot)(2)

RWC (%) = (FW − DW) × 100/(TW − DW)(3)

#### 4.4.2. Determination of Root Oxidative Activity

The α-naphthylamine oxidation method [63] was employed to determine the root activity. One gram of fresh root tissue was placed in a conical flask containing 50.00 mL of α-naphthylamine (α-NA) solution at 20 μg·L^−1^, and then incubated on a shaker at 25 °C for 3 h. After filtration, 2.00 mL of the filtrate was added to a test tube containing 1.00 mL of sodium nitrite, mixed well, and the absorbance at OD_510_ was measured. The oxidation amount of α-naphthylamine per gram of fresh root tissue per hour (μg·h^−1^·g^−1^ FW) was then calculated.

#### 4.4.3. Determination of Photosynthetic Pigment Content and Photosynthetic Gas Exchange Parameters

The extraction method [64] was employed for this phase of testing. Freshly chopped rice seedling leaves (0.10 g) were transferred into test tubes containing 10 mL of anhydrous ethanol and allowed to soak in the dark until the leaves were completely bleached, with five replications for each treatment. Using anhydrous ethanol as a blank, the absorbance at OD_470_, OD_649_, and OD_665_ was measured. The content of chlorophyll (Chl a, Chl b) and carotenoids (Car) (mg·g^−1^ FW) was calculated using the following formulas:

Chl a = 13.95 × OD_665_ − 6.88 × OD_649_ (4)

Chl b = 24.96 × OD_649_ − 7.32 × OD_665_ (5)

Total Chl = Chl a + Chl b(6)

Car = (1000 × OD_470_ − 2.05 × Chl a − 114.8 × Chl b)/245 (7)

Between 9:00 and 11:30 on the day of sampling, the net photosynthetic rate (Pn), stomatal conductance (gsw), intracellular carbon dioxide concentration (Ci), and transpiration rate (Tr) were measured using an LI-6800 portable photosynthesis system (LI-6800, LI-COR, Lincoln, NE, USA) [65]. The water use efficiency (WUE), stomatal limitation (Ls), and apparent leaf mesophyll conductance (AMC) were calculated using the following formulas:

WUE = Pn/Tr(8)

Ls = (Ca − Ci)/Ca × 100(9)

AMC = Pn/Ci(10)

#### 4.4.4. Determination of Reactive Oxygen Species and Membrane Damage Indices

The nitro-blue tetrazolium (NBT) method [66] was used for the histochemical staining of superoxide anion radicals (O_2_^·−^) in rice leaf tissues. From each experimental group, the initial fully developed leaf at the base was consistently harvested, with an analogous portion excised and submerged in phosphate-buffered saline (PBS, pH 7.80) containing 1% NBT (m/v). After vacuum infiltration, the samples were kept in the dark for 24 h until blue spots appeared. The samples were then transferred into a container with 85% ethanol and decolorized in a water bath at 80 °C until the leaves became bleached, after which they were observed and photographed.

The H_2_O_2_ content of the samples was determined using potassium iodide spectrophotometry [64], with 0.50 g of fresh samples weighed, ground in 5 mL of 0.1% TCA solution, and then subjected to centrifugation at 19,000× *g* for 20 min. The resulting supernatant was then added to a 10 mmol L^−1^ PBS solution with 1 mol L^−1^ potassium iodide solution, and a dark reaction was conducted at 28 °C for 1 h. The H_2_O_2_ content (μmol·g^−1^ FW) was then measured. The OD_390_ was employed to calculate the H_2_O_2_ content (μmol·g^−1^ FW). The MDA content was determined using the thiobarbituric acid (TBA) method, as described in a previous study [64]. Fresh samples (0.50 g) were weighed, ground in 10 mL of a 50 mmol L^−1^ PBS solution, and then centrifuged at 6000× *g* for 20 min. The resulting supernatant was transferred to a new tube and 0.6% TBA solution was added. The samples were then boiled in a water bath for 15 min. Following centrifugation at 4000× *g* for 20 min, the supernatant was collected to measure OD_450_, OD_532_, and OD_600_, with the aim of calculating the MDA content (mmol·g^−1^ FW).

Freshly cut rice leaves (0.10 g) were accurately weighed and placed in a beaker containing 10.00 mL of ultrapure water, and then fully soaked for 24 h. The electrical conductivity of the solution (EC1) was measured using a conductivity meter. Subsequently, the solution was boiled for 30 min, cooled to room temperature, and the electrical conductivity (EC2) was measured again. The relative electrical conductivity (REC) was calculated using the formula [64]:

REC (%) = (EC1/EC2) × 100.(11)

#### 4.4.5. Determination of Antioxidant System

A quantity of 0.50 g of fresh plant samples was weighed and put into an ice bath. A quantity of 10.00 mL PBS (pH 7.80) was added, grinding was conducted at 4 °C for homogenization, and 10,000× *g* centrifugation was carried out for 20 min to obtain the crude enzyme solution to be measured. The SOD enzyme activity was determined using the NBT method, as described in a previous study [67]. The enzyme extract was prepared, and riboflavin and NBT were added. The OD_560_ was then measured after illumination. The total SOD activity (U^−1^·g^−1^ FW) was calculated by taking 50% inhibition of NBT photochemical reduction by SOD activity units, with one enzyme activity unit (U) defined as the amount of enzyme required to inhibit 50% of the photochemical reduction of NBT. The POD enzyme activity was determined by the guaiacol method [68], whereby the catalysis by POD of the oxidation of guaiacol by H_2_O_2_ results in a product with maximum light absorption at 470 nm. The POD activity (U^−1^·g^−1^ FW) was calculated by measuring OD_470_ with a change of 0. CAT enzyme activity was determined by the UV-absorbent method [64], whereby H_2_O_2_ had maximum light absorption at 240 nm. CAT was thus able to decompose H_2_O_2_, which resulted in a decrease in the solution’s absorbance. CAT activity (U^−1^·g^−1^ FW) was calculated by measuring the change in OD_240_ per unit time [64].

The contents of AsA, DHA, GSH, and GSSG were determined by referring to the method proposed by Zhao [39], with minor modifications. AsA and red phenanthroline can form a chelate with a strong absorption peak at 534 nm, and the content of AsA can be calculated by determining the OD_534_ (μmol·g^−1^ FW). DHA is reduced to AsA by dithiothreitol (DTT), and the content of DHA can be calculated by determining the AsA production rate (μmol·g^−1^ FW). GSH and 2-nitrobenzoic acid (DTNB) can form compounds with strong absorption peaks at 412 nm, and the content of GSH can be calculated by determining the OD_412_ (μmol·g^−1^ FW). GSSG can be reduced to GSH by GR, and then reacted with DTNB, which allows for the determination of the OD_412_. APX catalyzes the oxidation of AsA. To calculate APX enzyme activity (U^−1^·g^−1^ FW), a decrease of 0.01 in OD_290_ in 1 min was taken as 1 U. The action of GR consumes reduced coenzyme II (NADPH), which has an absorption peak at 340 nm. Therefore, GR enzyme activity (U^−1^·g^−1^ FW) can be calculated by determining the change in OD_340_ (U^−1^·g^−1^ FW). The absorption peak of NADPH occurs at 340 nm, thus enabling the calculation of GR activity through the measurement of the change in OD_340_ (U^−1^·g^−1^ FW). DHAR is responsible for the production of 2-nitro-5-mercaptobenzoic acid (TNB) from GSH; thus, the activity of DHAR can be calculated by measuring OD_412_ (U^−1^·g^−1^ FW). The action of MDHAR results in a depletion of the NADH content. The MDHAR enzyme activity can be calculated by determining the rate of change in OD_340_ (U^−1^·g^−1^ FW).

#### 4.4.6. Determination of Osmotic Adjustment Substances

Proline (Pro) levels were assayed employing the sulfosalicylic acid technique [69]. Pro and sulfosalicylic acid can form a compound with a strong absorption peak at 520 nm, and the Pro content (μg·g^−1^ FW) can be calculated by measuring OD_520_. The determination of soluble protein (SP) content was carried out by using the Coomassie brilliant blue G-250 staining method [70]; SP and Coomassie brilliant blue form a compound with a strong absorption peak at 595 nm, and the SP content can be calculated by measuring OD_595_ (mg·g^−1^ FW).

#### 4.4.7. Determination of Endogenous Hormone Contents

The determination of endogenous hormones abscisic acid (ABA), cytokinin (CTK), gibberellin (GA), indole-3-acetic acid (IAA), jasmonic acid (JA), and salicylic acid (SA) was performed using assay kits developed by Shanghai Enzyme-Linked Biotech Co., Ltd. (Shanghai, China) The samples were analyzed using an enzyme-linked immunosorbent assay (ELISA) with a double-antibody one-step sandwich method, and the OD_450_ value was determined by an enzyme labeler (RT-6100) to calculate the sample concentration.

#### 4.4.8. Ion Content Determination

Fresh root and leaf samples were subjected to a 105 °C heat treatment for the purpose of killing any microorganisms. They were then transferred to a constant temperature oven set at 80 °C until a constant weight was achieved. The samples were ground into a homogeneous powder and weighed with an accuracy of 0.20 g for pre-treatment procedures such as disintegration and ashing. The concentrations of Na^+^, Cl^−^, K^+^, Ca^2+^, and Si^4+^ were determined through inductively coupled plasma mass spectrometry (ICP-OES, Thermo Scientific ICAP 6000 Series, Waltham, MA, USA).

#### 4.4.9. Observation of Rice Leaf Cell and Chloroplast Ultrastructure by Scanning Electron Microscopy and Transmission Electron Microscopy

Samples were taken after 7 d of NaCl treatment, the topmost fully expanded leaves of rice seedlings from each treatment group were excised and observed for surface changes using SEM (MIRA3, TESCAN, Europe, Brno, Czech Republic). A transmission electron microscope was used to further observe and analyze the ultrastructural changes in chloroplasts induced by nano-silica under NaCl stress conditions [71]. The collected leaf samples were fixed in a 0.10 mol·L^−1^ PBS (pH 7.00) solution containing 2.50% glutaraldehyde and left to soak for 24 h. The leaves were then rinsed with buffer solution and further fixed in a PBS solution containing 1% osmium tetroxide (m/v, OsO_4_). Following this, the specimens underwent dehydration via a serial dilution of ethanol (30%, 50%, 70%, 90%, 100%), and some samples were freeze-dried for 24 h. These samples were then observed using SEM (gold sputtering for 30 s, acceleration voltage of 5.00 kV), and images were captured. The remaining samples were embedded in epoxy resin, and ultra-thin sections of 70 to 90 nm thickness were cut using an ultramicrotome. These sections were then placed onto copper grids, stained, and observed using a TEM to analyze and collect images.

### 4.5. Statistical Analyses

Each parameter was analyzed using three technical replicates (*n* ≥ 3), with three technical replicates conducted for each experiment. The results are expressed as mean ± standard error (SEM), and the presence of different lowercase letters indicates a significant difference (*p* < 0.05). Data were organized using Microsoft Excel 2016 software. Statistical analysis, including one-way ANOVA, was performed using IBM SPSS Statistics 24 software. Graphs and charts were created using Origin 2019 and Microsoft PowerPoint 2016 software.

## 5. Conclusions

**Figure 12:**
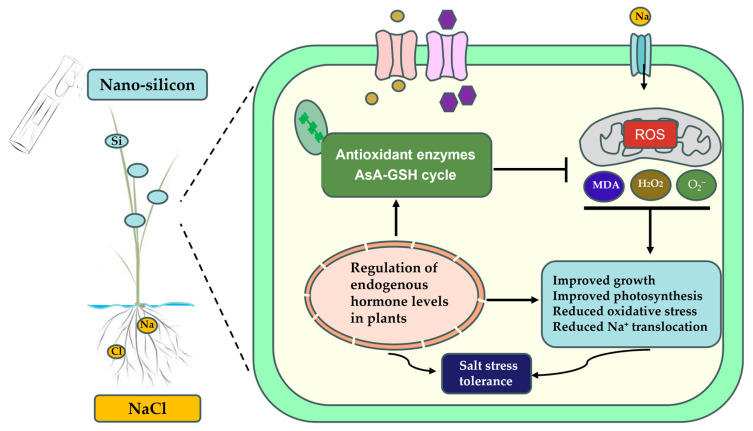
Modeling the response mechanism of nano-silicon to mitigate salt damage in rice seedlings.

In summary, the foliar application of nano-silicon can improve the coordinated growth, biomass accumulation, and photosynthetic efficiency of the root and above-ground parts of rice seedlings under NaCl stress. Nano-silicon also enhances the antioxidant defense system, balances Pro and SP levels, maintains the dynamic balance of Na^+^/K^+^, increases the uptake of nutrients, mitigates NaCl-induced oxidative damage, maintains the integrity of the chloroplasts and cell membranes, and jointly regulates the levels of endogenous hormones to alleviate the salt damage of rice seedlings and improve salt tolerance. The existence of complex but efficient regulatory mechanisms of nano-silicon in mitigating the negative effects of NaCl stress indicates the great potential of exploiting it in practical production, which could advance the development of environmentally friendly agricultural production (Figure 12). In addition, a deeper understanding of the molecular mechanisms behind the exogenous nano-silicon mitigation of salt damage in rice is more helpful in protecting and promoting practical production.

## Figures and Tables

**Table 1:** Effects of nano-silicon on morphological indicators of above-ground parts of rice seedlings under NaCl stress.

Treatment	Morphological Indicators				
	Shoot Length (cm)	Stem Diameter (mm)	Leaf Area (mm^2^)	Shoot Fresh Weight (×10^−2^ g)	Shoot Dry Weight (×10^−2^ g)
CK	32.46 ± 0.10 a	6.58 ± 0.05 a	872.47 ± 28.09 a	94.80 ± 1.82 a	17.96 ± 0.58 b
N	32.88 ± 0.58 a	6.74 ± 0.02 a	794.41 ± 29.65 a	99.18 ± 3.24 a	19.60 ± 0.72 a
S	28.74 ± 0.10 c	5.18 ± 0.10 c	575.51 ± 7.31 c	64.94 ± 0.84 c	13.80 ± 0.39 d
SN	30.10 ± 0.04 b	5.86 ± 0.14 b	688.46 ± 31.34 b	74.16 ± 1.10 b	15.88 ± 0.31 c

Note: Values represent mean *±* SEM (*n* ≥ 3). Control (CK), nano-silica treatment (N), salt treatment (S), and nano-silica + salt treatment (SN). CK vs. S indicates increase/decrease in S treatment compared to CK. S vs. SN indicates increase/decrease in SN treatment compared to S treatment. Different lowercase letters indicate significant difference at the 0.05 level among different treatments based on Duncan’s multiple range test.

**Table 2:** Effects of nano-silicon on root morphological indexes of rice seedlings under NaCl stress.

Treatment	Morphological Indicators		
	Root Length (cm)	Root Fresh Weight (×10^−2^ g)	Root Dry Weight (×10^−2^ g)
CK	20.18 ± 0.34 a	32.82 ± 1.32 a	3.98 ± 0.13 a
N	20.70 ± 0.58 a	33.74 ± 1.53 a	4.18 ± 0.10 a
S	17.78 ± 0.43 b	22.86 ± 0.59 c	2.56 ± 0.07 c
SN	19.58 ± 0.13 a	27.30 ± 0.89 b	3.22 ± 0.02 b

Different lowercase letters indicate significant difference at the 0.05 level among different treatments based on Duncan’s multiple range test.

**Table 3:** Effects of nano-silicon on root architecture of rice seedlings under NaCl stress.

Treatment	Morphological Indicators					
	Total Root Length (cm)	Root Surface (cm^2^)	Root Volume (cm^3^)	Root Diameter (×10^−2^ mm)	Root Bifurcation Number	Root Tips Number
CK	288.27 ± 49.07 ab	29.03 ± 3.23 b	0.55 ± 0.04 ab	43.34 ± 0.31 a	481.00 ± 26.15 b	634.33 ± 42.19 b
N	361.63 ± 31.15 a	39.44 ± 3.38 a	0.71 ± 0.08 a	43.97 ± 1.16 a	732.67 ± 58.95 a	927.00 ± 90.28 a
S	150.16 ± 4.62 c	12.93 ± 1.01 c	0.17 ± 0.06 c	32.08 ± 2.21 b	231.33 ± 35.15 c	271.00 ± 16.52 c
SN	253.22 ± 6.59 b	25.96 ± 0.50 b	0.43 ± 0.04 b	41.74 ± 1.60 a	396.33 ± 31.47 b	502.33 ± 17.53 b

Different lowercase letters indicate significant difference at the 0.05 level among different treatments based on Duncan’s multiple range test.

**Table 4:** Effects of nano-silicon on photosynthetic gas exchange parameters of rice seedlings under NaCl stress.

Treatment	Photosynthetic Gas Exchange Parameter						
	Pn (mmol·m^−2^s^−1^)	Tr (µmol·^−2^s^−1^)	Ci (µmol·mol^−1^)	Gs (mmol·m^−2^s^−1^)	WUE (mg·kg^−1^)	Ls (×10^−2^)	AMC (×10^−2^)
CK	17.42 ± 1.24 a	5.02 ± 0.28 a	302.94 ± 4.11 b	346.94 ± 13.02 a	628.99 ± 19.96 ab	22.14 ± 0.97 c	4.57 ± 0.15 a
N	17.64 ± 0.70 a	4.12 ± 0.28 b	316.75 ± 4.83 a	332.98 ± 38.39 a	683.45 ± 62.59 a	24.21 ± 0.93 c	4.26 ± 0.05 b
S	10.59 ± 0.28 c	2.13 ± 0.12 d	258.24 ± 2.21 d	94.70 ± 2.64 c	430.99 ± 11.41 c	35.08 ± 0.56 a	3.64 ± 0.03 c
SN	13.47 ± 0.88 b	3.00 ± 0.17 c	281.84 ± 2.76 c	179.93 ± 5.03 b	551.05 ± 7.64 b	32.32 ± 0.29 b	4.00 ± 0.04 b

Note: Pn, net photosynthetic rate. Tr, transpiration rate. Ci, intercellular CO_2_ concentration. Gs, stomatal conductivity. WUE, water use efficiency. Ls, stomatal limitation. AMC, apparent leaf mesophyll conductance. Different lowercase letters indicate significant difference at the 0.05 level among different treatments based on Duncan’s multiple range test.

**Table 5:** Phytoendogenous hormone contents in leaves and roots of rice seedlings.

Treatment		Endogenous Auxin					
		IAA (×10^−3^ nmol·g^−1^ FW)	CTK (ng·g^−1^ FW)	GA (pmol·g^−1^ FW)	ABA (ng·g^−1^ FW)	SA (ng·g^−1^ FW)	JA (pmol·g^−1^ FW)
Leaf	CK	404.30 ± 11.04 b	319.89 ± 9.50 b	1.12 ± 0.02 b	545.81 ± 9.17 c	44.44 ± 0.43 d	12.96 ± 0.16 d
	N	457.10 ± 1.96 a	429.41 ± 9.18 a	1.32 ± 0.02 a	456.89 ± 15.10 d	52.47 ± 0.71 c	14.89 ± 0.31 c
	S	264.00 ± 4.45 d	228.70 ± 7.97 c	0.73 ± 0.02 d	802.96 ± 10.51 a	59.06 ± 1.13 b	16.30 ± 0.19 b
	SN	322.30 ± 6.11 c	299.30 ± 7.61 b	1.00 ± 0.02 c	678.13 ± 15.43 b	69.35 ± 1.31 a	19.92 ± 0.44 a
Root	CK	485.40 ± 4.29 b	298.12 ± 6.54 b	0.91 ± 0.02 b	695.15 ± 12.660 c	37.45 ± 0.76 d	11.63 ± 0.28 b
	N	514.00 ± 3.42 a	359.22 ± 8.84 a	1.04 ± 0.03 a	548.13 ± 6.77 d	47.84 ± 0.98 c	15.41 ± 0.13 a
	S	327.10 ± 8.57 d	182.19 ± 2.10 c	0.75 ± 0.02 c	908.46 ± 8.73 a	55.21 ± 0.74 b	10.66 ± 0.39 b
	SN	406.00 ± 6.84 c	284.46 ± 8.18 b	0.77 ± 0.03 c	788.26 ± 11.86 b	61.10 ± 0.85 a	14.50 ± 0.38 a

Different lowercase letters indicate significant difference at the 0.05 level among different treatments based on Duncan’s multiple range test.

**Table 6:** Ion content in leaves and roots of rice seedlings.

Treatment		Rice Seedling Ion Content					
		Na^+^ (μg/g)	Cl^−^ (μg/g)	Si^4+^ (μg/g)	K^+^ (μg/g)	Ca^2+^ (μg/g)	Na^+^/K^+^ (×10^−3^)
Leaf	CK	321.20 ± 0.75 c	1462.02 ± 64.01 c	188.65 ± 3.30 c	8970.17 ± 14.25 a	1501.88 ± 1.07 b	35.80 ± 0.14 c
	N	127.06 ± 0.23 d	433.62 ± 25.34 d	367.92 ± 6.96 a	8666.60 ± 304.47 a	1833.36 ± 30.44 a	14.70 ± 0.54 d
	S	749.05 ± 2.60 a	4990.51 ± 73.55 a	124.54 ± 1.45 d	7448.48 ± 129.32 b	1419.38 ± 10.13 c	100.60 ± 1.40 a
	SN	650.04 ± 24.06 b	3830.46 ± 329.07 b	216.51 ± 10.45 b	7961.65 ± 143.74 b	1531.60 ± 7.31 b	81.60 ± 1.55 b
Root	CK	161.38 ± 0.55 c	428.27 ± 9.55 c	83.21 ± 0.16 c	2331.08 ± 10.65 a	214.88 ± 1.78 b	69.20 ± 0.08 c
	N	150.70 ± 0.16 c	262.43 ± 8.97 d	109.57 ± 1.30 a	2362.18 ± 94.98 a	239.54 ± 5.45 a	64.00 ± 2.51 c
	S	837.68 ± 0.56 a	1137.89 ± 20.41 a	63.93 ± 1.07 d	1368.44 ± 22.56 b	113.64 ± 3.65 c	612.50 ± 10.51 a
	SN	778.04 ± 11.89 b	1091.88 ± 3.95 b	101.83 ± 0.23 b	1493.62 ± 5.25 b	207.05 ± 1.30 b	521.00 ± 9.79 b

Different lowercase letters indicate significant difference at the 0.05 level among different treatments based on Duncan’s multiple range test.

## Data Availability

Data are contained within the article or Supplementary Materials.

## Supplementary Materials

The following supporting information can be downloaded at: https://www.mdpi.com/article/10.3390/ijms26010085/s1.
