# Supplementary material for: Mitigation Effect of Exogenous Nano-Silicon on Salt Stress Damage of Rice Seedlings
Source: Int J Mol Sci. 2024 Dec 25;26(1):85. doi: 10.3390/ijms26010085 (PMC11720159; doi:10.3390/ijms26010085)
Supplement: Supplementary file 1 [file ijms-26-00085-s001.zip › ijms-3342550-supplementary.pdf]

Mitigation Effect of Exogenous Nano-Silicon on Salt Stress Damage of Rice Seedlings

Jian Xiong [1,2], Xiaohui Yang [1,2], Minmin Sun [1,2], Jianqin Zhang [1,2], Linchong Ding [1,2], Zhiyuan Sun [1], Naijie Feng [1,2], Dianfeng Zheng [1,2] and Liming Zhao [1], Xuefeng Shen [1,2,*]

[1] College of Coastal Agricultural Sciences, Guangdong Ocean University, Zhanjiang 524008, China; 2112204010@stu.gdou.edu.cn (J.X.); 2112204049@stu.gdou.edu.cn (X.Y.); 2112204037@stu.gdou.edu.cn (M.S.); 21122040151@stu.gdou.edu.cn (J.Z.); dinglinchong0405@163.com (L.D.); sunzhiyuan@hndx2.wecom.work (Z.S.); fengnj@gdou.edu.cn (N.F.); zhengdf@gdou.edu.cn (D.Z.); nxyzlm@gdou.edu.cn (L.Z.)

[2] National Saline-Tolerant Rice Technology Innovation South China Center, Zhanjiang 524008, China

* Correspondence: shenxuefeng@gdou.edu.cn

Supplementary Materials:

TEM was used to observe the morphology of the nano-silica. which was found to be approximately spherical (Figure S1. A, B). The particle size distribution indicated that the particle diameter of the nano-silica material ranged from 12 to 17 nm (Figure S1. C). Energy-dispersive X-ray spectroscopy (EDS, Oxford Ultimmax, UK) was utilized to determine the elemental composition of the samples (Figure S1. D).

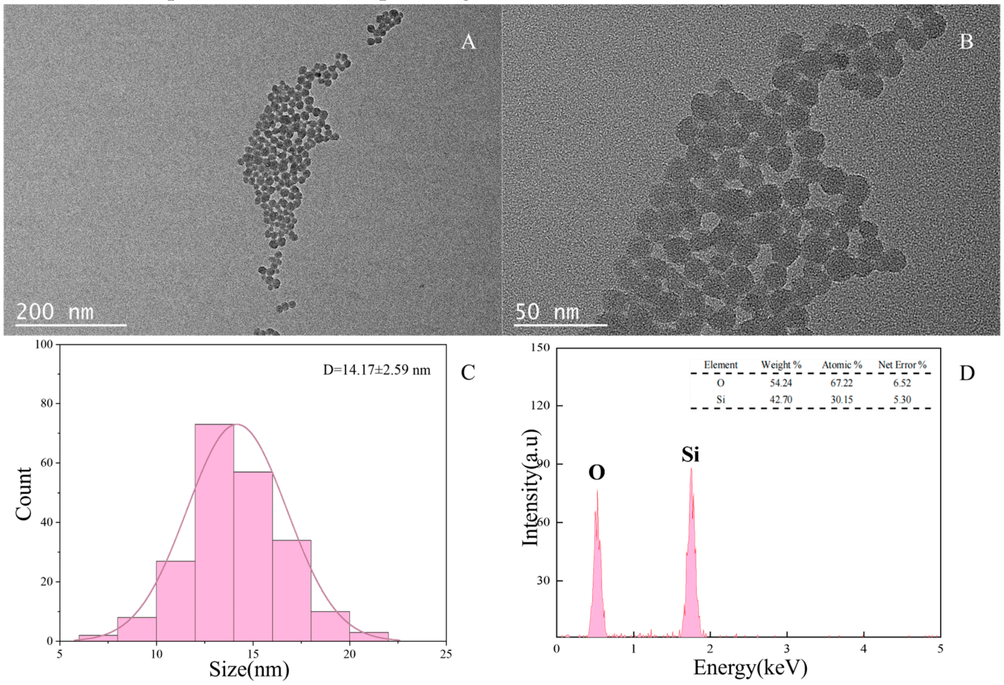

Figure S1. TEM of nano-silicon material (A, B); the particle size distribution (C); EDS energy spectrum analysis (D).

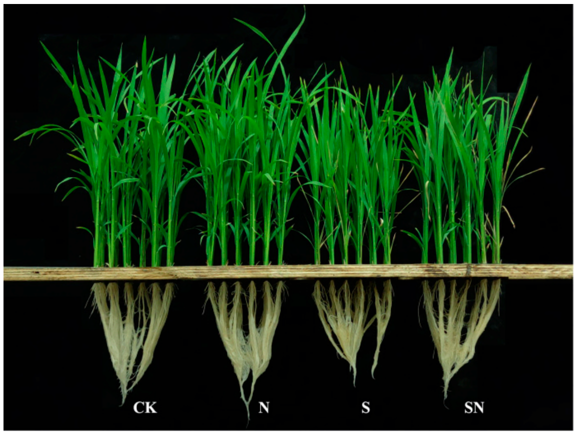

Figure S2. Effects of nano-silicon on the growth of rice seedlings under NaCl stress. (Images were acquired 7 d after NaCl treatment. Control (CK), nano-silica treatment (N), salt treatment (S), and nano-silica + salt treatment (SN)).

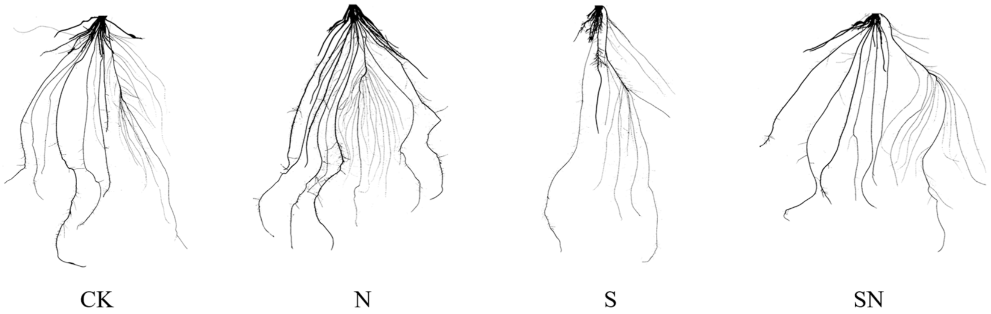

Figure S3. Effects of nano-silicon on root architecture of rice seedlings under NaCl stress. (Images were acquired 7 d after NaCl treatment. Control (CK), nano-silica treatment (N), salt treatment (S), and nano-silica + salt treatment (SN))

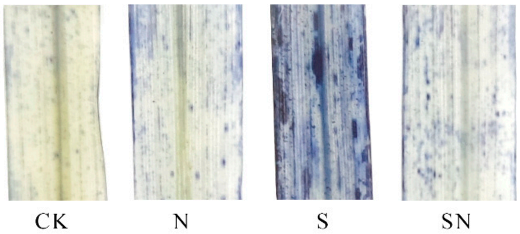

Figure S4. Effects of nano-silicon on $O_2^{\cdot-}$ distribution in leaves of rice seedlings under NaCl stress. (Control (CK), nano-silica treatment (N), salt treatment (S), and nano-silica + salt treatment (SN). Images were acquired 7 d after NaCl treatment.)

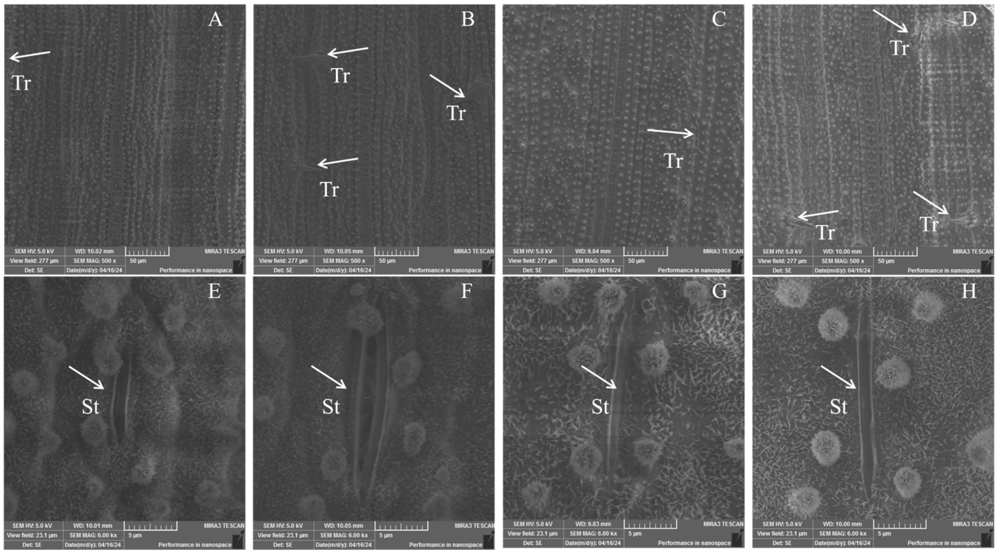

Figure S5. Scanning electron microscope observation on leaf surface of rice seedlings. (Control (CK), nano-silica treatment (N), salt treatment (S), and nano-silica + salt treatment (SN). CK (A and E), N (B and F), S (C and G), SN (D and H). Tr, trichome; St, stomatal apparatus.)

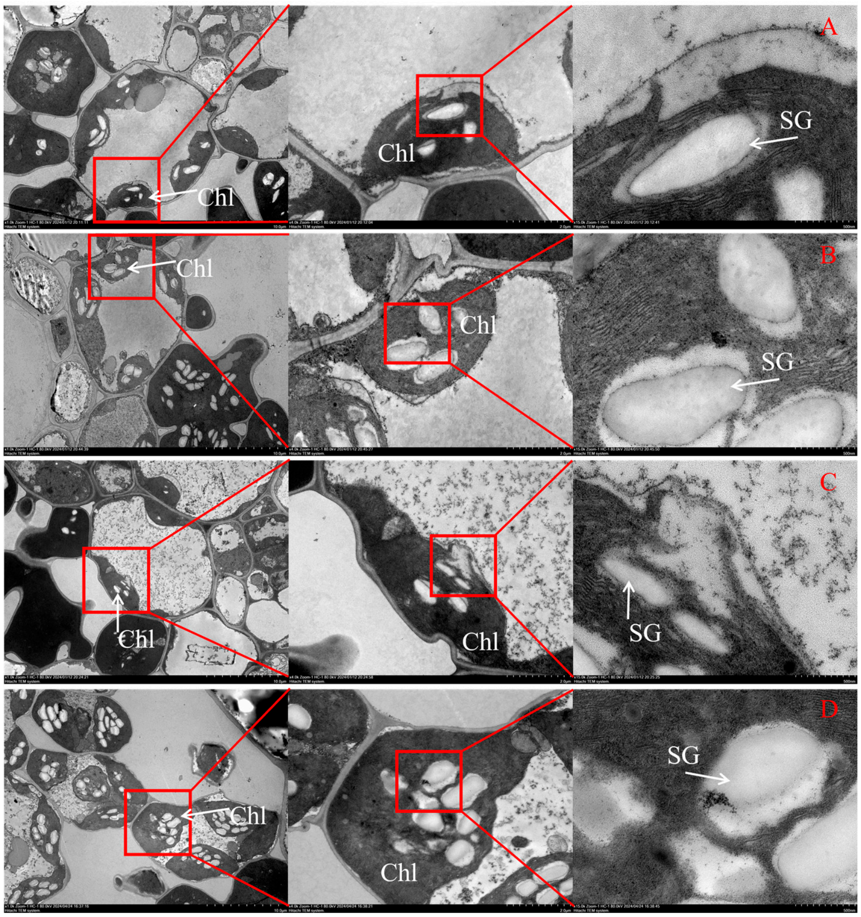

Figure S6. Observation of rice seedling leaf by transmission electron microscope. (Control (CK), nano-silica treatment (N), salt treatment (S), and nano-silica + salt treatment (SN). CK, (A); N, (B); S, (C); SN, (D). Chl, chloroplast; SG, starch grain.)

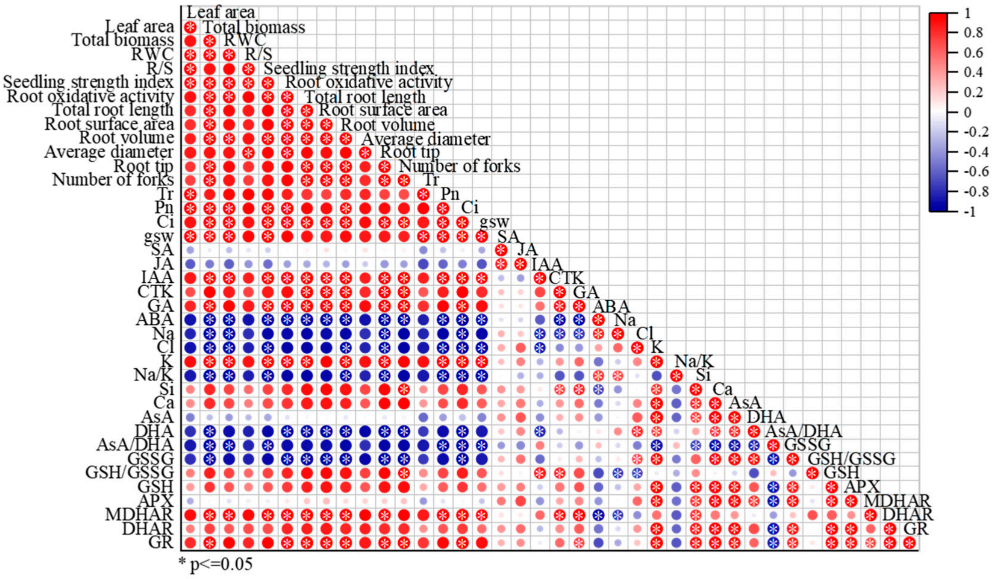

Figure S7. Pearson's correlation between seedling growth and physiological parameters in 9311 rice. Red colour indicates the positive correlation between these two parameters. Blue colour indicates negative correlation between these two parameters. SA, JA, IAA, CTK, GA, ABA, Na, Cl, K, Na/K, Si, Ca, AsA, DHA, AsA/DHA, GSSG, GSH/GSSG, GSH, APX, MDHAR, DHAR, and GR, respectively, represent the salicylic acid, jasmonic, indoleacetic acid, cytokinin, gibberellin, abscisic acid, Na^+ content, Cl^- conntent, K^+ conntent, Na^+/K^+ rate, Si^{4+} conntent, Ca^{2+} conntent, ascorbic acid, dehydroascorbic acid, AsA/DHA rate, glutathione(oxidized), GSH/GSSG rate, glutathione, ascorbate peroxidase, monodehydroascorbate enzyme, dehydroascorbate enzyme, glutathione reductase.
*** indicating significant at 5% levels of probability.**

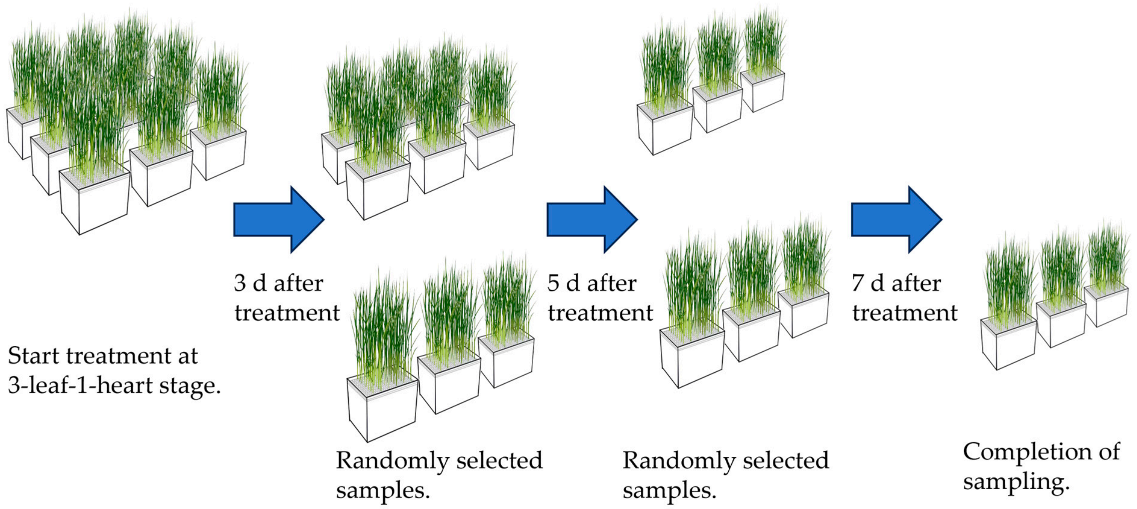

Figure S8. Schematic diagram of the experimental design. (A total of four treatments, CK, N, S and SN, with one treatment as a representative for the schematic design of the experiment, three boxes were randomly selected for sampling at 3 and 5 d post-treatment, respectively, and 20 seedlings with uniform growth within the treatments were selected for morphometric measurements after 7 d, with the remainder for sampling.)

Table S1. Two-way analysis of different treatments and sampling times of rice seedlings.

Indicators		MDA	H_2O_2	Pro	SP	SOD	POD	CAT	APX	MDHAR	DHAR	GR
Leaf	ST	24.02**	37.12**	369.88**	1.16^{ns}	11.83**	49.59**	5.14*	4.24*	8.19**	103.40**	107.39**
	Treat	5.95**	184.49**	164.67**	19.86**	1359.23**	75.85**	215.13**	94.66**	75.29**	139.07**	41.76**
	ST*Treat	41.65**	21.85**	30.74**	2.15^{ns}	11.83**	11.58**	8.12**	0.27^{ns}	2.23^{ns}	8.23**	8.62**
Root	ST	52.47**		43.27**	375.93**	0.78*	4.89*	8.546**	2.45^{ns}	0.60^{ns}	26.81**	3.47*
	Treat	7.00**		58.78**	146.23**	4.19*	96.57**	23.355**	35.61**	31.32**	26.11**	49.09**
	ST*Treat	33.67**		10.86**	67.31**	8.14**	2.42^{ns}	1.074^{ns}	1.56^{ns}	0.93^{ns}	0.94^{ns}	2.03^{ns}

ST, sampling time. Treat, treatment.

ns, *, and ** indicating non-significant and significant at 5% and 1% levels of probability, respectively.

Table S2. Nano-silicon concentration screening test data. (The treatments in the pre-test were carried out at the 2-leaf, 1-heart stage.).

Treatment	Pattern indicator							
	**Shoot length (cm)**	**Root length (cm)**	**Stem diameter (mm)**	**Leaf area (mm^2)**	**Shoot fresh weight ($\times 10^{-2}$g)**	**Root fresh weight ($\times 10^{-2}$g)**	**Shoot dry weight ($\times 10^{-2}$g)**	**Root dry weight (×10-2g)**
CK	25.60±0.31a	21.52±1.03a	3.72±0.04a	905.26±20.04a	43.96±0.61a	21.36±0.74a	8.69±0.11a	2.14±0.09a
S	22.32±0.13d	17.98±0.29d	2.92±0.12d	617.20±15.73d	35.68±0.63c	15.10±0.39c	6.09±0.12d	1.64±0.05b
SN1	23.04±0.32c	18.38±0.31cd	3.04±0.04cd	662.24±4.07cd	37.40±0.40c	16.80±0.95bc	6.20±0.25cd	1.71±0.05b
SN2	23.38±0.25c	17.66±0.20cd	3.08±0.08cd	653.52±16.99cd	37.40±1.03c	17.22±0.83bc	6.73±0.10bc	1.83±0.08b
SN3	23.68±0.17bc	18.50±0.47bc	3.20±0.08bc	716.92±27.01bc	38.48±0.48bc	17.70±0.82b	7.16±0.10b	1.83±0.10b
**SN4**	**24.12±0.15b**	**19.28±0.19b**	**3.34±0.07b**	**784.48±42.31b**	**40.82±1.43b**	**18.94±1.00b**	**7.30±0.08b**	**1.86±0.06b**
SN5	23.46±0.20bc	17.64±0.47d	2.90±0.04d	712.02±35.85bc	36.96±1.54c	17.80±0.24b	6.34±0.37cd	1.77±0.13b

Note: Values represent mean ± SEM (n ⩾ 3). Control (CK), NaCl treatment(S), foliar application of nano-silicon 0.25 mmol·L^{-1} + NaCl treatment (SN1), foliar application of nano-silicon 0.50 mmol·L^{-1} + NaCl treatment (SN2), foliar application of nano-silicon 1.00 mmol·L^{-1} + NaCl treatment (SN3), foliar application of nano-silicon 2.00 mmol·L^{-1} + NaCl treatment (SN4), foliar application of nano-silicon 4.00 mmol·L^{-1} + NaCl treatment (SN5).Different lowercase letters indicate significant difference at 0.05 level among different treatments based on Duncan's multiple range test. The same below.
